# Genetic variation and exercise-induced muscle damage: implications for athletic performance, injury and ageing

**DOI:** 10.1007/s00421-016-3411-1

**Published:** 2016-06-13

**Authors:** Philipp Baumert, Mark J. Lake, Claire E. Stewart, Barry Drust, Robert M. Erskine

**Affiliations:** Research Institute for Sport and Exercise Sciences, Liverpool John Moores University, Liverpool, L3 3AF UK

**Keywords:** Exercise-induced muscle damage, Delayed onset muscle soreness, Single nucleotide polymorphism, Creatine kinase, Elderly

## Abstract

Prolonged unaccustomed exercise involving muscle lengthening (eccentric) actions can result in ultrastructural muscle disruption, impaired excitation–contraction coupling, inflammation and muscle protein degradation. This process is associated with delayed onset muscle soreness and is referred to as exercise-induced muscle damage. Although a certain amount of muscle damage may be necessary for adaptation to occur, excessive damage or inadequate recovery from exercise-induced muscle damage can increase injury risk, particularly in older individuals, who experience more damage and require longer to recover from muscle damaging exercise than younger adults. Furthermore, it is apparent that inter-individual variation exists in the response to exercise-induced muscle damage, and there is evidence that genetic variability may play a key role. Although this area of research is in its infancy, certain gene variations, or polymorphisms have been associated with exercise-induced muscle damage (i.e. individuals with certain genotypes experience greater muscle damage, and require longer recovery, following strenuous exercise). These polymorphisms include *ACTN3* (R577X, rs1815739), *TNF* (−308 G>A, rs1800629), *IL6* (−174 G>C, rs1800795), and *IGF2* (ApaI, 17200 G>A, rs680). Knowing how someone is likely to respond to a particular type of exercise could help coaches/practitioners individualise the exercise training of their athletes/patients, thus maximising recovery and adaptation, while reducing overload-associated injury risk. The purpose of this review is to provide a critical analysis of the literature concerning gene polymorphisms associated with exercise-induced muscle damage, both in young and older individuals, and to highlight the potential mechanisms underpinning these associations, thus providing a better understanding of exercise-induced muscle damage.

## Introduction

People who engage in unaccustomed, strenuous physical exercise can experience stiff or sore muscles, a feeling that is usually apparent for 24–72 h after exercise. This phenomenon is known as delayed onset muscle soreness. Several investigations have revealed that these unaccustomed eccentric actions, during which the muscle is lengthened while it is active, provoke stiffer and more tender muscles compared to concentric or isometric contractions (Armstrong 1984; Armstrong et al. 1991). These contractions are strongly associated with damage to skeletal muscle consisting of structural disruption of sarcomeres, disturbed excitation–contraction coupling and calcium signalling, leading to an inflammatory response and the activation of several muscle protein degradation pathways. This process has been referred to as exercise-induced muscle damage (Hyldahl and Hubal 2014; Peake et al. 2005) and is normally accompanied by swelling, and a temporary reduction in both maximum strength and range of motion (Baird et al. 2012; Brown et al. 1999; Clarkson et al. 1992). Circulating muscle-specific proteins [e.g., creatine kinase (CK), myoglobin and α-actin] are commonly used to indicate exercise-induced muscle damage (Huerta-Alardín et al. 2005; Martinez Amat et al. 2007), whereas tenascin-C is thought to be an indicator for disruption of the overlying connective tissue and the extracellular matrix (Flück et al. 2003).

Exercise-induced muscle damage can be divided into the initial damage phase, which occurs during the exercise bout, and the secondary damage phase, which is linked with the delayed inflammatory response (Kuipers 1994; Howatson and Van Someren 2008). These phases are eventually followed by muscle remodelling (Flann et al. 2011; Thiebaud 2012; Tidball 2005). Although there is evidence to suggest that a certain amount of muscle damage is a positive stimulus for muscle restructuring, hypertrophy and strength gains (Roig et al. 2008), in rare cases, strenuous unaccustomed exercise can lead to exertional rhabdomyolysis, which is characterised by muscle fibre necrosis (Warren et al. 2002b). Intracellular muscle contents leak into the circulation and extracellular fluid, which can lead to kidney failure or even to death (Knochel 1990; Clarkson et al. 2005b). Furthermore, the response to muscle damage seems to be age-dependent. There is evidence to suggest that older people are more susceptible to muscle damage compared to young adults, which is reflected by impaired muscle regeneration and hampered remodelling (Conceição et al. 2012; Peake et al. 2010; Snijders et al. 2009).

From the plethora of studies that have investigated exercise-induced muscle damage, it is apparent that variability in the response to muscle damaging exercise exists between (Vincent et al. 2010; Clarkson et al. 2005b) and within studies (Nosaka and Clarkson 1996). Variations between studies can occur due to different study population, age, gender and a small sample size (Eynon et al. 2013; Toft et al. 2002). However, intra-study variation within a homogenous cohort warrants further consideration, with evidence to suggest that genetic variability may play a role. Some genes have common variations in sequence, known as polymorphisms, which, depending on where this polymorphism occurs within the gene, can directly affect gene expression and ultimately the amount of protein produced. The most common type of sequence variation is a single nucleotide polymorphism (SNP), where one nucleotide substitutes another. Another type of common sequence variation is the insertion/deletion (indel) polymorphism, in which a specific nucleotide sequence is present (insertion) or absent (deletion) from the allele. Some polymorphisms can modify the protein product, thus potentially altering function. It follows, therefore, that polymorphisms of genes encoding key proteins in the muscle–tendon unit (such as the *ACTN3* R577X SNP) have implications for the ability to recover from strenuous exercise, thus influencing the risk of injury. This may be particularly relevant in elite athlete groups, who are known to have different genetic profiles compared to the general population (Yang et al. 2003; Myerson et al. 1999). Moreover, specific gene polymorphisms (e.g. *COL1A1* rs1800012, *COL5A1* rs12722, rs3196378, *MMP3* rs679620, rs591058 and rs650108) have been associated with tendon/ligament injury prevalence (e.g., Achilles tendinopathy/rupture and anterior cruciate ligament rupture) (Bell et al. 2012; Laguette et al. 2011; Collins and Raleigh 2009). However, very little is known about the potential genetic association with muscle damage and muscle regeneration in response to muscle damaging exercise, either in young or older people, or the mechanisms that underpin that association.

As older people appear to be more susceptible to exercise-induced muscle damage than younger adults (Jiménez-Jiménez et al. 2008; Manfredi et al. 1991; Fielding et al. 1991; Roth et al. 2000), older people with a genetic predisposition to greater muscle damage, may be at a greater risk of developing muscle–tendon unit injury (Laguette et al. 2011; September et al. 2007). As a result, these individuals may experience prolonged disuse and therefore increased ageing-associated muscle atrophy (i.e., sarcopaenia), which is associated with reductions in strength and quality of life. Knowing who requires longer to recover from a bout of strenuous exercise, may help practitioners prescribe personalised exercise medicine to their patients, thus optimising health and reducing the risk of injury and further muscle wasting. One of the greatest challenges facing exercise genetic research is the investigation of functionally relevant genetic variation and of their mechanisms of action. The aims of this review are to (1) provide a critical review of the current literature on exercise-induced muscle damage and, therefore, to improve our understanding of the different phases of the responses to muscle damaging exercise; (2) emphasise those studies that have investigated the association between genetic variation and muscle damage, both in young and older people; and (3) propose mechanistic explanations that may underpin these associations.

## Genetic variation and the initial phase of exercise-induced muscle damage

**Fig. 1:**
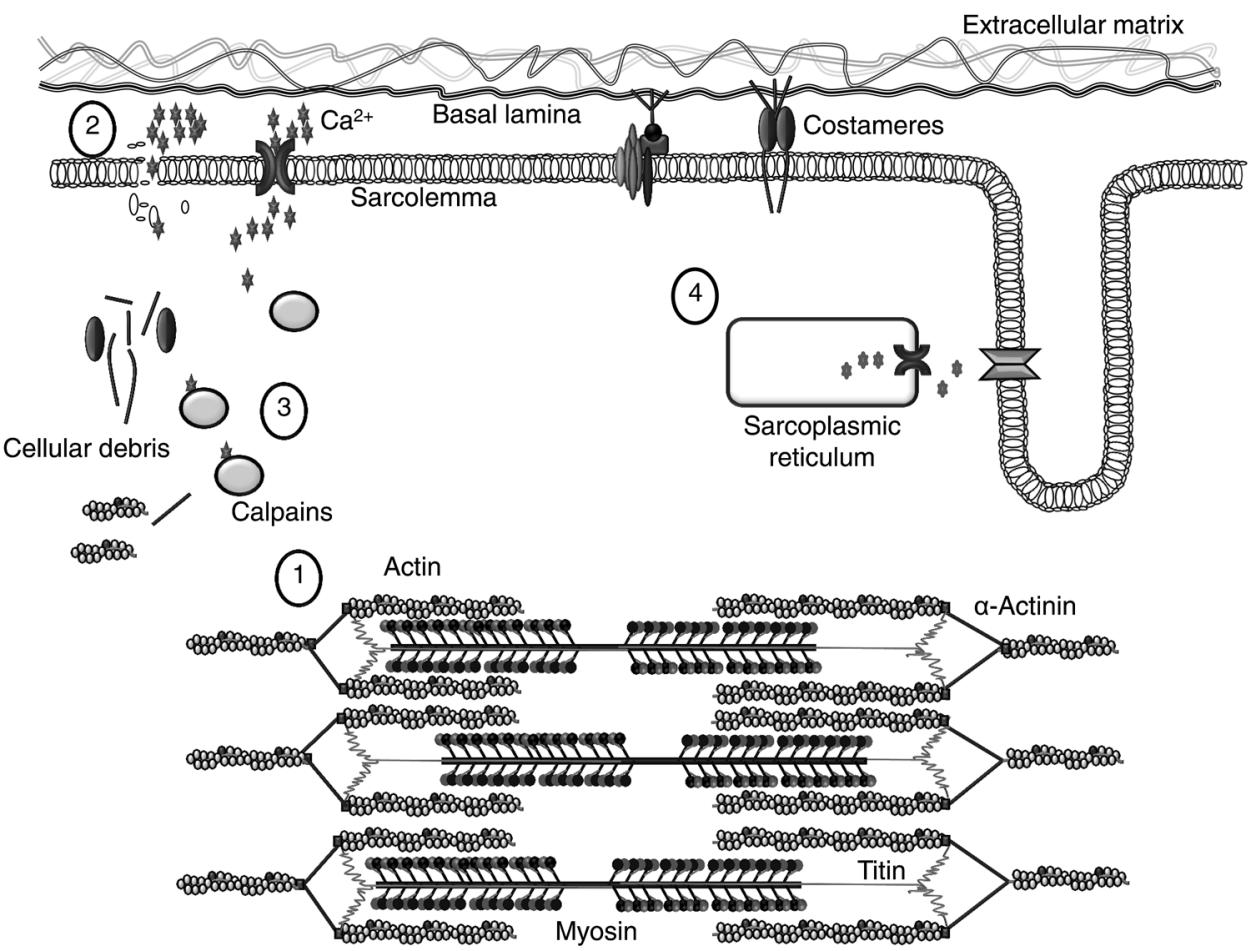
Initial phase of exercise-induced muscle damage. Due to different abilities of each sarcomere to resist eccentric actions, some of the sarcomeres will be stretched beyond the optimum overlap of actin and myosin filaments, resulting in Z-line streaming (Morgan, 1990) (*1*). This is accompanied by increased permeability of the sarcolemma (*2*). Extracellular Ca^2+^ influx into the muscle fibre activates different Ca^2+^-sensitive proteases (calpains). Calpain activation leads to proteolysis of cytoskeletal and costameric proteins (Thiebaud 2012) (*3*). However, a failure of excitation–contraction coupling also seems to play an important role in strength loss following strenuous exercise, as murine muscle exposed to caffeine revealed an attenuated loss of muscle strength (Warren et al. 1993) (*4*). Figure adapted from Hyldahl and Hubal (2014)

Exercise-induced muscle damage can result in damage to the ultrastructure of the muscle fibre (including Z-line streaming), to the extracellular matrix, and to overextended sarcomeres and t-tubules of skeletal muscle tissue (Brown et al. 1997b; Kjær 2004; Friden and Lieber 1992, 2001; Friden et al. 1981). Structural disruption of sarcomeres is thought to be caused by the heterogeneity of sarcomere length (Morgan 1990) and, consequently, some sarcomeres resist eccentric actions more than others (Allen et al. 2005; Friden et al. 1981). Prolonged strain causes weaker sarcomeres to be stretched beyond the optimum overlap of actin and myosin filaments (Fig. 1). This results in popped sarcomeres and appears as a broadening, smearing or even disruption of the Z-lines. Interestingly, the thinnest Z-lines are detected in the faster (type II) muscle fibres, which generate the highest shortening velocities, and the widest Z-lines are found in slow (type I) muscle fibres (Knoll et al. 2011). Consequently, fast-twitch fibres are more sensitive than slow twitch fibres to Z-disk streaming (Proske and Morgan 2001; Appell et al. 1992). This mechanical damage is one mechanism by which a prolonged loss of strength occurs immediately after excessive strain (Cheung et al. 2003; Hyldahl and Hubal 2014; Friden and Lieber 1992).

The transmission of muscle fibre force to the tendon (leading to joint movement) occurs not only in the longitudinal direction in line with the direction of pull of the tendon, but also in the lateral direction (between adjacent fibres to the overlying connective tissue and extracellular matrix) (Kjær 2004; Hughes et al. 2015). The extracellular matrix in skeletal muscle provides structural and biochemical support to the contractile tissue, and is associated with the inflammatory response and satellite cell activation (see “Skeletal muscle remodelling following exercise-induced muscle damage”) (Hyldahl and Hubal 2014; Kjær 2004). The relative proportion of different collagen subtypes in the extracellular matrix of skeletal muscle and tendon varies depending on the position and function of the connecting tissues (Kjær 2004; Duance et al. 1977; Davis et al. 2013). The contractile apparatus is connected to the extracellular matrix by costameres (structural complexes comprising proteins such as dystrophin, focal adhesion kinase and integrins) and by intermediate filament proteins, such as desmin (Hughes et al. 2015). According to Ramaswamy et al. (2011), more than 80 % of muscle force is transferred via this lateral pathway. Thus, costameres, intermediate filament proteins and the extracellular matrix are considered essential for the integrity of skeletal muscle and the maintenance of lateral force transmission. Furthermore, they are thought to play an important role in injury prevention by stabilising the myofilaments (Lovering and De Deyne 2004; Stauber et al. 1990; Hughes et al. 2015). The degradation of cytoskeletal, costameric and extracellular matrix proteins could negatively influence the lateral transmission of force between adjacent muscle fibres, which could, at least in part, be the source of the prolonged decrease of maximum strength seen following strenuous exercise (Raastad et al. 2010).

Activation of Ca^2+^ proteases (calpains) appears to play an important part in the muscle damage–repair process. Damage to the sarcolemma results in the accumulation of excess intracellular Ca^2+^, which activates different calcium-sensitive proteases, localised predominantly at the I band and Z disk regions of myofibrils (Belcastro et al. 1998). The activation results in proteolysis within minutes of cytoskeletal and costameric proteins (Thiebaud 2012; Lovering and De Deyne 2004; Boppart et al. 2008; Zhang et al. 2008; Allen et al. 2005), and calpain activity is still measurable three days after exercise-induced muscle damage (Raastad et al. 2010). This intra- and extracellular damage requires the removal and repair of the damaged proteins, and is therefore followed by an inflammatory response and by activation of the ubiquitin–proteasome pathway (see “Genetic variation and the secondary phase of exercise-induced muscle damage”) (Wei et al. 2005; Tidball 2005). However, the loss of strength after eccentric muscle contractions was reversed by exposing murine muscle to caffeine (Balnave and Allen 1995; Warren et al. 1993). Caffeine facilitates the influx of free intracellular Ca^2+^ from sarcoplasmic reticulum into the cytosol of the muscle (Warren et al. 1993; Proske and Morgan 2001). This phenomenon cannot be explained by damage to the sarcomere, so it can be concluded that sarcomere damage is not the only cause of strength loss, as impaired ECC also appears to play a role (Cheung et al. 2003; Hyldahl and Hubal 2014). Increased permeability of the sarcolemma, due to damaged muscle fibre structure, metabolic disturbance, and fibre remodelling, is likely to be the main reason for elevated plasma CK and myoglobin (Kjær 2004; Baird et al. 2012).

A repeated bout of the same eccentric exercise causes significantly fewer symptoms, such as a lower sensation of pain and almost no increase in serum CK activity plus faster recovery of muscle function (Brown et al. 1997a). This well-established phenomenon is referred to as the repeated bout effect and can last up to six months (Nosaka et al. 2001). A repeated bout of strength training results in a different expression of genes, which are involved in pro- and anti-inflammatory responses, leading to reduced inflammation (Gordon et al. 2012). There is also evidence that the repeated bout effect, at least in part, is based on restructuring of the muscle after damage (McHugh 2003). Likewise, extracellular matrix remodelling is believed to be associated with protection of skeletal muscle against future damage, which is indicated by an increase in gene expression of collagen types I and III and laminin-β2 (Mackey et al. 2011). This is thought to occur in line with muscle remodelling of intermediate filaments and the addition of sarcomeres in series (leading to longer fibres) (Friden et al. 1984; Armstrong 1990; Hyldahl and Hubal 2014).

Considering all of the above, candidate SNPs influencing the initial phase of contraction-induced damage are likely to be functional SNPs of genes encoding key structural proteins within the sarcomere, the extracellular matrix and the costameric protein complexes linking the two. The following sections will highlight the evidence to support this hypothesis. Table 1 summarises every candidate SNP that has been discussed in this review.

**Table 1:** Gene polymorphisms associated with exercise-induced muscle damage

Gene polymorphism	Subjects	Exercise performed	‘Protective’ allele	References
*ACE* (I/D) (rs4646994)	Moderately active young men and women	50 unilateral eccentric elbow flexion contractions	D	Yamin et al. (2007)
	Physically active young men and women	Step up exercise for 5 min followed by 15 knee bends with a backpack weighted at 30 % of their body weight	–	Heled et al. 2007
*ACTN3* R577X (rs1815739)	Untrained healthy young men and women	50 unilateral eccentric elbow flexion contractions	–	Clarkson et al. (2005b)
	Untrained healthy young men	4 series of 20 bilateral maximal eccentric knee extensions	R	Vincent et al. (2010)
	Wild type and *Actn3* knockout mice	Eccentric contractions on isolated extensor digitorum longus muscles at 30 % stretch	R	Seto et al. (2011)
	Professional male soccer athletes	Plyometric leg exercise	R	Pimenta et al. (2012)
	Moderately active young men	Two bouts of 50 drop jumps separated by two weeks	X	Venckunas et al. (2012)
	Male and female patients	Retrospective cohort study for risk of exertional rhabdomyolysis	R	Deuster et al. (2013)
	Female athletes	Retrospective cohort study for risk of muscle injury	X	Iwao-Koizumi et al. (2014)
*CCL2* −3441(C>T) (rs3917878)	Untrained healthy young males and females	50 unilateral eccentric elbow flexion contractions	C	Hubal et al. (2010)
*CCL2* −289 (G>C) (rs2857656)	Elite soccer players	Retrospective cohort study for risk of non-contact musculoskeletal soft tissue injuries	C	Pruna et al. (2013)
*CCR2* −941(A>C) (rs3918358)	Healthy untrained men and women	50 unilateral eccentric elbow flexion contractions	A	Hubal et al. (2010)
*CCR2* 4439 (T>C) (rs1799865)	Healthy untrained men and women	50 unilateral eccentric elbow flexion contractions	T	Hubal et al. (2010)
*CKM* Ncol (A>G) (rs1803285)	Moderately active young men and women	Step up exercise for 5 min followed by 15 knee bends with a backpack weighted at 30 % of their body weight	G	Heled et al. (2007)
	Moderately active young men and women	50 unilateral eccentric elbow flexion contractions	–	Yamin et al. (2010)
	Healthy men and women of different ages	4–21 km running race	–	Miranda-Vilela et al. (2012)
	Male and female patients	Retrospective cohort study for risk of exertional rhabdomyolysis	A	Deuster et al. (2013)
*IGF2* 13790 (C>G) (rs3213221)	Healthy untrained men and women	50 unilateral eccentric elbow flexion contractions	C	Devaney et al. (2007)
*IGF2* 17200 (G>A) (rs680)	Healthy untrained men and women	50 unilateral eccentric elbow flexion contractions	G	Devaney et al. (2007)
*IGF2AS* 1364 (A>C) (rs4244808)	Healthy untrained men and women	50 unilateral eccentric elbow flexion contractions	C	Devaney et al. (2007)
*IGF2AS* 11711 (G>T) (rs7924316)	Healthy untrained men and women	50 unilateral eccentric elbow flexion contractions	G	Devaney et al. (2007)
*IL1B* −3737 (C>T) (rs4848306)	Healthy untrained men	3 sets of 8 contractions at 80 % of the subject’s maximal voluntary contraction followed by a 4th set to voluntary failure for leg press, leg curl, and leg extension, respectively	C	Dennis et al. (2004)
*IL1B* −511 (C>T) (rs16944)	Healthy untrained men	3 sets of 8 contractions at 80 % of the subject’s maximal voluntary contraction followed by a 4th set to voluntary failure for leg press, leg curl, and leg extension, respectively	–	Dennis et al. (2004)
	(Non-) professional athletes versus control	Cross-sectional study	–	Cauci et al. (2010)
*IL1B* 3954 (C>T) (rs1143634)	Healthy untrained men	3 sets of 8 contractions at 80 % of the subject’s maximal voluntary contraction followed by a 4th set to voluntary failure for leg press, leg curl, and leg extension, respectively	T	Dennis et al. (2004)
	(Non-) Professional athletes versus control	Cross-sectional study	–	Cauci et al. (2010)
*IL6* −174 (G>C) (rs1800795)	Moderately active young men and women	50 unilateral eccentric elbow flexion contractions	G	Yamin et al. (2008)
	Male and female patients	Retrospective cohort study for risk of exertional rhabdomyolysis	–	Deuster et al. (2013)
	Older obese women	7 sets of 10 bilateral eccentric knee extensions with a load corresponding to 110 % of 10-repetition maximum.	C	Funghetto et al. (2013)
*INS* 1045 (C>G) (rs3842748)	Healthy untrained men and women	50 unilateral eccentric elbow flexion contractions	C	Devaney et al. (2007)
*MLCK* 49 (C>T) (rs2700352)	Untrained healthy young men and women	50 unilateral eccentric elbow flexion contractions	C	Clarkson et al. (2005b)
*MLCK* 37885 (C>A) (rs28497577)	Untrained healthy young men and women	50 unilateral eccentric elbow flexion contractions	C	Clarkson et al. (2005b)
	Male and female patients	Retrospective cohort study for risk of exertional rhabdomyolysis	C	Deuster et al. (2013)
*OPN* −66 (T>G) (rs28357094)	Healthy untrained men and women	24 unilateral eccentric elbow flexion contractions	T	Barfield et al. (2014)
*SLC30A8* (C>T) (rs13266634)	Untrained healthy young men and women	50 unilateral eccentric elbow flexion contractions	T	Sprouse et al. (2014)
*SOD2* (C>T) (rs4880)	Healthy male and female volunteers of different ages	4–21 km running race	C	Akimoto et al. (2010)
*TNF* −308 (G>A) (rs1800629)	Moderately active young men and women	50 unilateral eccentric elbow flexion contractions	A	Yamin et al. (2008)

### Alpha-actinin-3 R577X polymorphism and the initial phase of exercise-induced muscle damage

Of all the polymorphisms that have been associated with exercise-induced muscle damage, the most investigated is the *ACTN3* R577X SNP (Clarkson et al. 2005b; Deuster et al. 2013; Pimenta et al. 2012; Seto et al. 2011; Venckunas et al. 2012; Vincent et al. 2010) (Table 1). The protein isoforms, α-actinin-2 and α-actinin-3, are crucial components of the Z-line in mammalian skeletal muscle and anchor actin filaments to the Z-lines, cross-linking the thin filaments to the adjacent sarcomeres (Mills et al. 2001; North et al. 1999; Blanchard et al. 1989). Whilst α-actinin-2 is ubiquitously expressed in skeletal muscle, α-actinin-3 is only expressed in fast-twitch fibres of human skeletal muscle (North and Beggs 1996; North et al. 1999). A functional SNP (rs1815739; substitution of a C with a T nucleotide) results in an abortive stop codon (X-allele) rather than the expression of the amino acid arginine (R-allele) at amino acid 577 of exon 16 on chromosome 11, resulting in an individual being either RR, RX or XX genotype. As a consequence, XX homozygotes are not able to express the protein α-actinin-3 (MacArthur and North 2004; North and Beggs 1996; North et al. 1999). A sub-section of the population is XX homozygous, ranging from less than 1 % in African Bantus to 18 % in Europeans, to 25 % in Asian populations (Mills et al. 2001). Absence of α-actinin-3 does not result in a disease phenotype due to compensatory up-regulation of α-actinin-2 (North et al. 1999) but there is evidence that this nonsense SNP affects physical performance (Erskine et al. 2014; Niemi and Majamaa 2005; Clarkson et al. 2005a; Moran et al. 2007).

The *ACTN3* XX genotype has been associated with smaller muscle volume (Erskine et al. 2014), slower baseline sprint times (Niemi and Majamaa 2005; Moran et al. 2007), lower strength (Erskine et al. 2014; Clarkson et al. 2005a), and lower muscle power (Clarkson et al. 2005a; Seto et al. 2011; Walsh et al. 2008; Moran et al. 2007; Erskine et al. 2014). These findings are supported by *Actn3* knock-out mouse models, demonstrating a shift in the properties of fast muscle fibres towards a more oxidative fast fibre profile, lower muscle strength, reduced mass and decreased diameter of IIb fibres (Chan et al. 2011; MacArthur et al. 2007, 2008). Strong evidence has been presented that, as a consequence of the up-regulation of α-actinin-2 in XX homozygotes, more calsarcin-2 is bound to α-actinin-2 and less to calcineurin (Seto et al. 2013). The binding affinity of calsarcin-2, which functions as an inhibitor of calcineurin activation, is greater for α-actinin-2 compared to α-actinin-3. Consequently, a higher level of free calcineurin is able to activate the downstream signalling of the slow myogenic programme. Given the larger size, higher force and power generating capacity, and lower fatigue resistance of type II fibres compared to type I fibres (Bottinelli et al. 1996), the evidence presented by Seto et al. (2013) provides a mechanistic explanation for the associations between *ACTN3* genotype and muscle size, strength, power, and endurance phenotypes.

Recent investigations have suggested that α-actinin-3 may be evolutionarily optimised for the minimization of muscle damage (Yang et al. 2003). The majority of the human studies support the hypothesis that XX homozygotes are more susceptible to strenuous exercise compared to their RR or RX counterparts (Pimenta et al. 2012; Vincent et al. 2010; Deuster et al. 2013). For instance, *ACTN3* XX homozygotes are approximately three times more likely to develop exertional rhabdomyolysis compared to people of RR or RX genotypes (Deuster et al. 2013). However, other studies have revealed no differences between *ACTN3* genotypes regarding markers of muscle damage (Clarkson et al. 2005b), or have shown contrary effects post-exercise (Venckunas et al. 2012) or in muscle injury risk (Iwao-Koizumi et al. 2014). The cross-sectional study of Clarkson et al. (2005b) revealed no differences in strength loss but a lower baseline CK activity in the blood in *ACTN3* XX homozygotes compared to carriers of the *ACTN3* R-allele. These baseline differences in CK activity may have been due to *ACTN3* genotype-dependent differences in muscle mass (i.e., smaller muscle volume in XX homozygotes versus R-allele carriers) (Erskine et al. 2014).

**Fig. 2:**
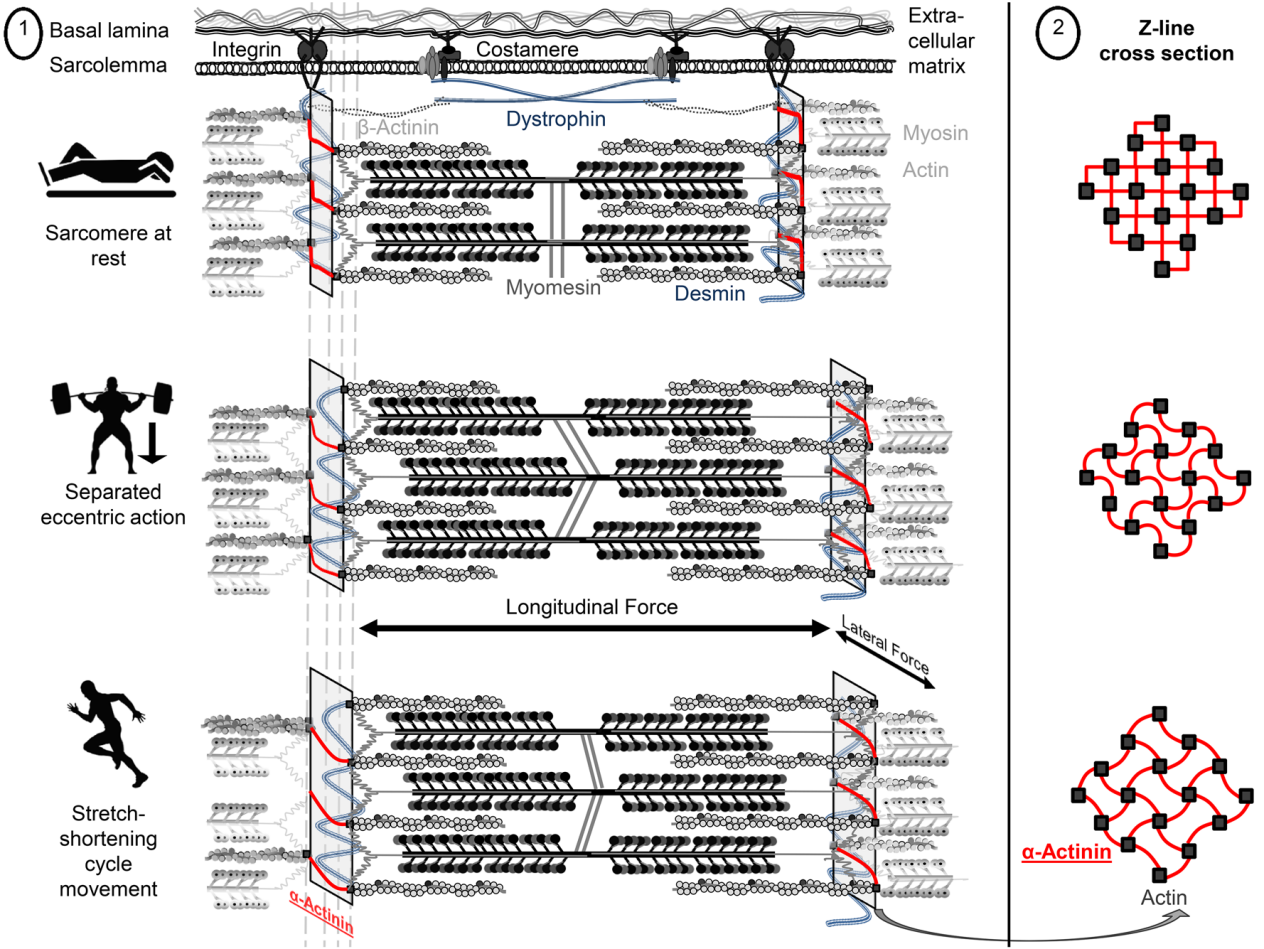
Proposed changes in sarcomere structure during stretch–shortening cycle movements and purely eccentric actions, focussing on α-actinin (highlighted in *red and underlined*). The *left-hand side* shows the sarcomere longitudinally in a quasi-3D model at rest, and the α-actinin elongation during purely eccentric actions, and stretch–shortening cycle movements (*1*). The *right-hand side* illustrates the sarcomere cross-section at the level of the Z-line (*2*). At rest, α-actinin is set to roughly 90° between the antiparallel actin filaments, while under active tension, the space between the antiparallel actin filaments increases and α-actinin is stretched to a basket-weave lattice (Gautel 2011). Alpha-actinin is thought to play a key role in the longitudinal (via the anchoring of actin filaments to the Z-line) and lateral (via costamere fibre-to-fibre interaction) transmission of muscle fibre force (Hughes et al. 2015; Yang and Xu 2012). Moreover, human type II muscle fibres from *ACTN3* XX homozygotes (where α-actinin-3 deficiency is compensated by the presence of α-actinin-2) are less stiff than type II muscle fibres from *ACTN3* R-allele carriers (Broos et al. 2012). Thus, it is likely that α-actinin-2 is able to store more energy than α-actinin-3 during the active stretch phase of the stretch–shortening cycle, which is released during the shortening phase (Kjær 2004; Yang and Xu 2012). We propose that stretch–shortening cycle movements increase the actin filament spacing to a greater extent compared to purely eccentric actions, thus elongating α-actinin to become almost completely straight at peak eccentric force. Individuals with α-actinin-3 deficiency (*ACTN3* XX homozygotes) might, therefore, benefit from having a more elastic Z-line during stretch–shortening cycle movements compared to R-allele carriers (Broos et al. 2012), resulting in a reduced damage response to stretch–shortening movements (Venckunas et al. 2012). Figure adapted from Gautel (2011) (color figure online)

Movements with repeated stretch–shortening cycles, eccentric followed by immediate concentric muscle contraction) (Venckunas et al. 2012) seem to have a different demand profile for the muscle–tendon unit compared to purely eccentric actions (Fig. 2) (Seto et al. 2011; Vincent et al. 2010). Due to the fact that α-actinin is linked to both the longitudinal and lateral transmission of force (Hughes et al. 2015; Yang and Xu 2012), we propose that α-actinin-3 deficiency (XX genotype) with a more elastic Z-line (Broos et al. 2012) might result in benefits to stretch–shortening cycle movements compared to R-allele carriers. Although stretch–shortening cycle includes an eccentric element, contrary to the type of maximal eccentric contractions typically used in exercise-induced muscle damage studies, the force and the eccentric phase involved in the active braking phase of stretch–shortening cycles are generally fast and of short duration (Nicol et al. 2006). Interestingly, muscle activation decreases with increasing velocity in the eccentric phase under the stretch–shortening cycle conditions (Benoit and Dowling 2006), which indicates that other non-contractile (elastic) structures, such as the extracellular matrix/tendon, might provide important contribution to the power output by storing energy (Kjær 2004; Yang and Xu 2012). Indeed, a highly compliant elastic musculotendinous system is thought to elevate the use of elastic strain energy in stretch–shortening cycle movements (Wilson et al. 1991). Thus, individually performed eccentric actions with greater longitudinal force transmission might damage the link between the contractile structure and the Z-line, which might activate the calpain system to a greater extent.

The transmission of muscle fibre force to the tendon may occur faster by the stiffer Z-line including α-actinin-3 in the longitudinal direction (Hughes et al. 2015; Broos et al. 2012) and, also, might reduce muscle damage in eccentric actions performed without a stretch–shortening cycle compared to the α-actinin-3 deficient fibres (Seto et al. 2011; Vincent et al. 2010). Head et al. (2015) revealed a significantly increased sarcoplasmic reticulum Ca^2+^ pumping and leakage in *ACTN3* XX homozygotes, which was probably due to a higher expression of the specific Ca^2+^ channel sarco(endo)plasmic reticulum calcium-adenosine-triphosphatase-1 gene, and of the Ca^2+^ binding proteins, calsequestrin and sarcalumenin, in the sarcoplasmic reticulum (Head et al. 2015). Increased dynamics with elevated intracellular Ca^2+^ levels during and after exertional muscle damage may lead to increased cytoskeletal damage and membrane disruption (Zhang et al. 2008; Head et al. 2015; Quinlan et al. 2010). Muscle damage induced by exclusively performed eccentric actions might lead to increased desmin degradation (Yu 2013), which results in fewer connections with the extracellular matrix and adjacent myofibrils, and could be an explanation for the higher susceptibility of XX homozygotes in this mode of exercise. Taken together, the different effect of the *ACTN3* R577X SNP in diverse mode of exercises could explain the fact that studies show mixed results. This may be why there are differences in *ACTN3* genotype frequency in short and long distance athletes of stretch–shortening cycle-related sports (e.g., running) (Yang et al. 2003), whereas both short and long distance athletes in power sports, commonly carried out without stretch–shortening cycles (e.g., swimming), show no difference in genotype/allele frequency distribution (Ben-Zaken et al. 2015). This demonstrates why future studies should not only distinguish between power and endurance athletes, but should focus on sport-specific movements when investigating the association with genetic variation.

### Myosin light chain kinase polymorphisms and the initial phase of exercise-induced muscle damage

Every myosin head is connected with two light chains on the long lever arm, which are known as the essential and the regulatory light chains. In skeletal and cardiac muscles of mammals, troponin and tropomyosin have the role of triggering the contraction following the increase in free cytosolic Ca^2+^, while the regulatory light chain modulates Ca^2+^ activation (Sweeney et al. 1993; Cheung et al. 2003; Lossie et al. 2014). Repeated Ca^2+^ influx due to muscular contraction activates myosin light chain kinase, and this enzyme phosphorylates the regulatory light chains. It has been shown that regulatory light chain phosphorylation results in increased Ca^2+^ sensitivity (Szczesna et al. 2002), which increases the rate of force development predominantly in type II muscle fibres (Childers and McDonald 2004). This might be the result of an increased number of force-generating cross-bridges. However, the increased force output by light chain phosphorylation might also result in elevated muscle damage, which has been shown in skinned fast-twitch fibres (Childers and McDonald 2004).

Two different SNPs of the myosin light chain kinase gene [49 (C>T) (rs2700352) and 37885 (C>A) (rs28497577)] have been investigated concerning exercise-induced muscle damage (Clarkson et al. 2005b). T-allele carriers of the 49 (C>T) SNP have shown increased baseline strength in comparison to CC carriers but TT homozygotes revealed increased circulatory levels of the muscle damage biomarkers (CK and myoglobin) following eccentric exercise. Furthermore, A-allele carriers of the 37885 (C>A) SNP have revealed greater muscle strength loss and increased plasma CK following strenuous exercise. This is in line with the findings of Deuster et al. (2013), who showed that exertional rhabdomyolysis cases are about five times more likely for the A-allele of the 37885 (C>A) SNP of the myosin light chain kinase gene compared to carriers of the C-allele. The mechanisms, however, are unclear. Clarkson et al. (2005b) suggested that these SNPs may alter regulatory light chain phosphorylation, thus leading to higher muscle strain and subsequently greater muscle damage following strenuous exercise.

### Muscle-specific creatine kinase polymorphisms and the initial phase of exercise-induced muscle damage

The creatine kinase enzyme is expressed in the cytosol and mitochondria of tissues with high energy consumption (e.g., skeletal muscle fibres). The cytosolic enzyme is composed of the two subunits muscle type (M) and brain type (B), which provide three different combination possibilities: CK-BB (predominantly in brain), CK-MB (in cardiac muscle) and CK-MM (in skeletal muscle). Skeletal muscle-specific CK is bound to the M-line structure and to the sarcoplasmic reticulum of myofibrils (Wallimann et al. 1992; Brancaccio et al. 2007). In healthy individuals, most serum CK consists of skeletal muscle CK (Brancaccio et al. 2007). Creatine kinase can leak from muscle fibres into the circulation following the mechanical tearing of the sarcolemma and opening of stretch-activated channels following contraction-induced damage, although the exact mechanism is still unclear (Allen et al. 2005).

The skeletal muscle CK-encoding gene is located at the 19q13.2–13.3 region of the chromosome 19 (Nigro et al. 1987). The Ncol (A>G) SNP (rs1803285) of the muscle creatine kinase gene, is mapped to the 3′ untranslated region, which means it could affect the localization, translation efficiency and stability of the mRNA, which might mediate the location and function of the protein (Wilson et al. 1995). Interestingly, the genes for the ryanodine receptor 1 (Robinson et al. 2006) and myotonic dystrophy protein kinase (Brunner et al. 1989), which are associated with muscle function and specific myopathies, are mapped to the same area of chromosome 19. According to Deuster et al. (2013), Ncol GG homozygotes are present in 28.1 % of African Americans, in 14.2 % of Caucasians, 0 % of Hispanic and 8.3 % of Asian individuals. Investigations of the Ncol SNP of the muscle creatine kinase gene have revealed different outcomes. In the study of Deuster et al. (2013), GG homozygotes were reportedly 3.1 times more likely to experience exertional rhabdomyolysis than carriers of the A-allele. However, Heled et al. (2007) revealed that *NcoI* AA homozygotes had a sixfold higher risk of being a high responder of circulating CK to eccentric exercise than GG or AG genotypes. Other studies do not support a role for the Ncol SNP of the muscle creatine kinase gene in explaining the CK variability between individuals (Miranda-Vilela et al. 2012; Yamin et al. 2010). However, the mechanism remains poorly understood and is confounded by the different methodological designs implemented by researchers. Furthermore, Heled et al. (2007) and Yamin et al. (2010) have only investigated CK response as a marker for muscle damage. Further studies with several other muscle damage markers such as muscle strength loss and soreness could provide a better physiological/systems-based understanding of the influence of this NcoI SNP on exertional muscle damage. An additional restriction fragment length polymorphism, the TaqI SNP of the muscle creatine kinase gene, has been shown to be in strong linkage disequilibrium with the NcoI SNP (Miranda-Vilela et al. 2012). The TaqI 1-2 genotype has indicated a lower risk for inflammation after a track event between 4 and 21 km, whereby the participants could choose their preferential distance. However, no further studies have been undertaken towards understanding a potential role for this SNP in association with muscle damage. It is possible that these SNPs change the half-life of the CK enzyme and the intracellular concentration of CK (Heled et al. 2007). Elevated intracellular CK concentration might increase calpain activation, thus resulting in greater protein degradation.

## Genetic variation and the secondary phase of exercise-induced muscle damage

**Fig. 3:**
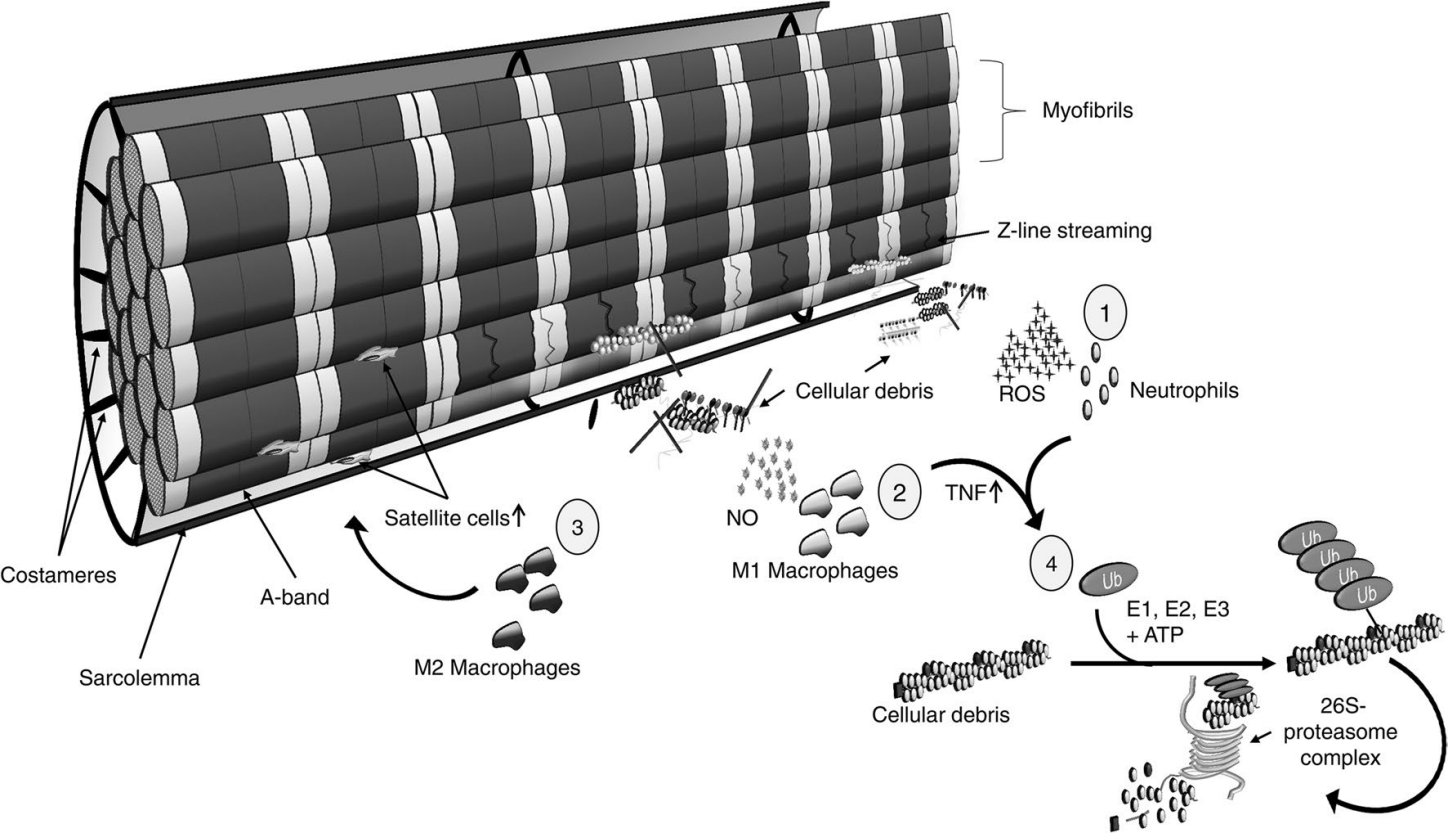
The secondary phase of muscle damage. Leucocytes infiltrate the site of myotrauma (Tidball 2005). Firstly, neutrophils migrate to damaged muscle fibres and produce reactive oxygen species (ROS) to degrade cellular debris (Suzuki et al. 1996) (*1*). Neutrophils are substituted by macrophages within 24 h (Malm et al. 2000), with M1 macrophages removing cellular debris by producing cytotoxic levels of nitric oxide (NO) (*2*). In the latter stage of muscle damage, a shift from M1 to M2 macrophages is associated with the activation of satellite cells and the subsequent regeneration of muscle fibres (Tidball 2011) (*3*). Neutrophils and macrophages also express tumour necrosis factor (TNF), which activates the ubiquitin–proteasome pathway (Tidball and Villalta 2010) (*4*). This pathway regulates proteolysis by attaching ubiquitin polymers (Ub) to cellular debris via three different types of enzymes (E1–E3 ligases). As a result, these ubiquitin-marked proteins will be degraded by the 26S-proteasome complex (Reid 2005)

The secondary phase of muscle damage is a complex event that has been linked to inflammation (Schoenfeld 2010), where leucocytes infiltrate muscles with damaged fibres and remain there for days or even weeks (Tidball 2005). Although the results of published studies are inconsistent (Schneider and Tiidus 2007), in vitro (Kanda et al. 2013; Suzuki and Ford 1999) and in vivo studies (Paulsen et al. 2010) support a role for neutrophils in muscle damage. It is assumed that neutrophils (Suzuki et al. 1996) migrate to the region of injury in the early stage of muscle damage (Fig. 3). Neutrophils contribute to the degradation of damaged muscle tissue by producing reactive oxygen species (ROS), which are reported to attract macrophages to the area of trauma (McGinley et al. 2009; Nguyen and Tidball 2003).

Reactive oxygen species can directly and indirectly modulate muscle damage through several mechanisms (Toumi et al. 2006). A potential mechanism to link oxidative stress with calpain-mediated proteases is via ROS decreasing plasma membrane Ca^2+^-adenosine-triphosphatase activity (Siems et al. 2003), which might encourage Ca^2+^ accumulation within the cell (Powers and Jackson 2008). Although ROS is toxic, it may also play an important role as a secondary messenger in cell signalling and in the regulation of gene expression resulting in ROS-mediated adaptation to exercise (Schoenfeld 2012; Hornberger et al. 2003; Crane et al. 2013).

In contrast to neutrophils, there is strong evidence that macrophages and monocytes infiltrate the endomysium and especially the perimysium of the injured area of the muscle (Hubal et al. 2008; Paulsen et al. 2010). Macrophages replace neutrophils within 24 h and remain present for up to 14 days after exercise (Malm et al. 2000). During the early stages of muscle damage, there is an increase of M1 macrophages (which express CD68 surface marker but not CD163), supporting the removal of cellular debris by producing cytotoxic levels of nitric oxide. This is followed by a shift from M1 to M2 macrophages (CD68^−^/CD163^+^), which promote the activation of satellite cells and the subsequent regeneration of muscle fibres (see “Skeletal muscle remodelling following exercise-induced muscle damage”) (Mahoney et al. 2008; Kanda et al. 2013; Tidball and Villalta 2010; Philippou et al. 2012; Zanou and Gailly 2013).

Leucocyte accumulation and the following remodelling appear to be gradual processes regulated by the extent of damage (Paulsen et al. 2010, 2012). In an extreme case of muscle damage, remodelling may become maladaptive characterised by necrosis, incomplete healing, and fibrotic scar tissue formation (Butterfield 2010). Cytokines play particularly well-characterised roles in an orchestrated regulated fashion of the activation and modulation of the inflammatory response (Paulsen et al. 2012). Recent investigations revealed that some cytokines are also expressed by skeletal muscle, and are therefore named myokines (Pedersen et al. 2003). The role of cytokines in the phase of inflammation following exercise-induced muscle damage is explained in the comprehensive review of Paulsen et al. (2012). Cytokines are classified as (1) pro-inflammatory cytokines [promoting inflammation, e.g. interleukin (IL)-1α, IL-1β and tumour necrosis factor (TNF)]; (2) anti-inflammatory cytokines (inhibiting inflammation, e.g. IL-10, IL-4 and IL-13) and chemokines (abbreviated from chemotactic cytokines), which attract leucocytes and other cells to migrate from the blood to the region of injury [e.g., chemokine (C–C motif) ligand 2 (CCL2)] (Paulsen et al. 2012; Peake et al. 2005; Suzuki et al. 2002). Muscle cytokine expression after strenuous exercise is predominantly pro-inflammatory (Peake et al. 2005).

In addition, some cytokines such as IL-6 can act either as a pro- or an anti-inflammatory agent, depending on the environment (Pedersen and Febbraio 2008). The majority of cytokines are released from several cell types including muscle fibres, fibroblasts, neutrophils, and macrophages, and the expression of cytokines is determined by the mode, intensity and duration of exercise (Peake et al. 2015). Furthermore, the action patterns of some of these cytokines change during the inflammatory response. These findings make it difficult to identify the specific roles of each cytokine after exercise-induced muscle damage (Smith et al. 2008). However, the invading neutrophils and macrophages express TNF at the early phase of inflammatory response (Philippou et al. 2012; Tidball and Villalta 2010; Warren et al. 2002a). Tumour necrosis factor is able to activate the ubiquitin–proteasome pathway, which is one of the main mechanisms for the cellular protein degradation in eukaryotic cells (Murton et al. 2008; Li et al. 2005). The ubiquitin–proteasome pathway regulates proteolysis by attaching ubiquitin polymers to damaged proteins via three distinct types of enzymes (known as E1–E3 ligases). Subsequently, the 26S-proteasome complex degrades the ubiquitin-marked protein (Reid 2005). Tumour necrosis factor increases the gene expression of the E3 ligases, muscle ring finger 1 (MuRF1) and muscle atrophy F-box (MAFbx; also referred to as Atrogin1) (Li et al. 2003, 2005; Murton et al. 2008; Bodine et al. 2001). Thus, it is thought that TNF is an important factor in the instigation of the remodelling process after exertional muscle damage (Murton et al. 2008).

There is evidence to suggest that muscles of older individuals exhibit higher levels of damage following strenuous exercise than of younger individuals (Jiménez-Jiménez et al. 2008; Manfredi et al. 1991; Fielding et al. 1991; Roth et al. 2000). Biopsies from the vastus lateralis muscle revealed greater muscle damage in older men in comparison to younger subjects immediately after eccentric exercise (Manfredi et al. 1991; Roth et al. 2000). Furthermore, older women demonstrated a threefold greater percentage reduction in strength 24 h after unaccustomed eccentric exercise than younger women (Roth et al. 2000). In addition, the recovery time to baseline strength was prolonged (up to 7 days) compared with the young sedentary subjects (4 days) (Ploutz-Snyder et al. 2001). Other studies support the finding that the secondary phase of exercise-induced muscle damage appears to differ between older and younger adults (Thalacker-Mercer et al. 2010; Jiménez-Jiménez et al. 2008). On closer examination, neutrophil (Cannon et al. 1994) and both M1 and M2 macrophage (Przybyla et al. 2006; Hamada et al. 2005) recruitment is impaired in muscle from older individuals in the secondary phase of muscle damage following strenuous exercise. The increase in plasma IL-6 concentration following eccentric exercise also tends to be blunted in older versus younger adults (Toft et al. 2002; Conceição et al. 2012). This is further supported by findings of blunted increases in muscle TNF and transforming growth factor-β1 (TGF-β1) and larger increase of IL-1β messenger ribonucleic acid (mRNA) expression within older muscle after eccentric exercise (Przybyla et al. 2006; Hamada et al. 2005). These findings could simply be due to the difficulty in raising levels pro-inflammatory cytokine levels over and above the chronically elevated levels found in older people (see “Skeletal muscle remodelling following exercise-induced muscle damage”). Alternatively, as macrophages are the major source of TNF and TGF-β1 within the muscle following exercise-induced muscle damage (Tidball 2011; Fadok et al. 1998), it is possible that lower macrophage recruitment in older individuals would lead to lower TNF and TGF-β1 expression and production (Hamada et al. 2005). Unaccustomed high-intensity resistance exercise (sufficient to cause moderate muscle damage) has been shown to induce greater nuclear factor kappa-light-chain-enhancer of activated B (NF-κB) and heat shock protein 70 protein expression in older versus younger human adult muscle (Thalacker-Mercer et al. 2010). Nuclear factor kappa-light-chain-enhancer of activated B is activated by pathways associated with muscle protein degradation (Roubenoff et al. 2003): its activation up-regulates the expression of muscle-specific ubiquitin ligases MAFbx and MuRF1 (Gumucio and Mendias 2013; Patel et al. 2014). Heat shock proteins mediate the correct folding of denatured proteins which would explain the increased expression of heat shock protein 70 in accordance with increased NF-κB activation following muscle damage (Thalacker-Mercer et al. 2010; Morton et al. 2009) (see “Genetic variation and the initial phase of exercise-induced muscle damage”). Given these compelling studies, the question arises as to whether cytokine SNPs also play a role in muscle damage induction or repair or both.

### Interleukin-1 polymorphisms and the secondary phase of exercise-induced muscle damage

The interleukin-1 (*IL1*) family of cytokine genes is located together on chromosome 2, and includes IL-1α (*IL1A*), IL-1β (*IL1B*) and IL-1 receptor antagonist (IL-1Ra; *IL1RN*) (Dennis et al. 2004). Interleukin-1α and IL-1β are agonists of the IL-1 receptor type I (IL-1R1) and promote inflammation. In general, IL-1β acts synergistically with TNF and induces the expression of several other pro-inflammatory genes (Dinarello 2009). Following eccentric exercise in humans, systemic levels of IL-1β increase marginally (Peake et al. 2005), but there is an increase of local IL-1β levels within skeletal muscle up to five days post exercise (Fielding et al. 1993). In contrast, IL-1Ra acts as an antagonist of IL-1R1, preventing the binding of IL-1α and IL-1β with IL-1R1, respectively. Instead of IL-1β, IL-1Ra is highly concentrated in plasma following intense physical exercise (Paulsen et al. 2012). In the absence of IL-1Ra, the activity of IL-1 is unrestricted and leads to increased inflammatory response (Dinarello 2009).

Different SNPs of the *IL1B gene* have been investigated in relation to the response to exercise and exercise-induced muscle damage: (1) at position −511 (C>T) (rs16944) in the promoter region (di Giovine et al. 1992); (2) at position +3954 (C>T) (rs1143634) in exon 5 (TaqI restriction site polymorphism) (Bioque et al. 1995); and (3) at position −3737 (C>T) (Dennis et al. 2004; Vangsted et al. 2011). Dennis et al. (2004) investigated the associations of selected *IL1* SNPs with the inflammatory response following a single bout of resistance exercise. Twenty-four sedentary Caucasian males were recruited based on specific clusters of *IL1* SNPs (haplotypes) (+4845 *IL1A*, +3954 *IL1B*, −511 *IL1B*, and −3737 *IL1B* polymorphisms). Only participants with the *IL1B* C/C (+3954) or with the T/T (−3737) genotype showed an increased inflammatory response (changes in inflammatory associated cytokines and M1 macrophages number) in skeletal muscle. However, the concentration of macrophages did not change. This leads to the assumption that the cytokine release by each macrophage is elevated or local production by the skeletal muscle itself is increased. Individuals with the above-mentioned genotypes, who also carried the C-allele of the *IL1RN* +2018 (T>C) SNP, demonstrated a further increase of inflammatory response following resistance exercise.

Cauci et al. (2010) found that the *IL1B* +3954 (C>T) SNP, together with the −511 (C>T), have no influence on athletic phenotype, which is in accordance with the findings that neither plasma IL-1β nor *IL1B* mRNA is influenced by physical activity (Petersen and Pedersen 2005; Mahoney et al. 2008). In addition, a multi-allelic insertion polymorphism in intron 2 of the *IL*-*1RN* gene (rs380092) contains a variable number tandem repeat of an 86-bp length of DNA (Mansfield et al. 1994). Allele 2 (two repeats of the 86 bp region) of the *IL1RN intron 2* variable number tandem repeat was significantly more frequent in athletes compared to non-athletes. In addition, there was a higher frequency distribution of the 1/2 (allele 1 with four repeats and allele 2 with two repeats of the 86 bp region) genotype variable number tandem repeat *IL1RN* in high-grade professional athletes than in non-professional athletes. In contrast, the frequency of *IL1RN* allele 2 homozygotes did not differ between athletes and non-athletes. Unfortunately, this study has only distinguished between professional (high-grade), non-professional (medium-grade) athletes, and non-athletes. Athlete status was not discriminated within the different types of sport, which is necessary, as different mode of exercises require different physical traits. However, in vitro investigations showed that the *IL1RN* allele 2 has been associated with a lower expression of IL-1Ra (Dewberry et al. 2000), but increased production of the pro-inflammatory cytokine IL-1β (Santtila et al. 1998). Cauci et al. (2010) suggested that carriers of *IL1RN* allele-2 displayed a moderate increase of IL-1-dependent inflammation, which results in benefits to athletic performance. *IL1RN* allele 2 might support the removal of cellular debris, promoting a faster recovery. However, *IL1RN* allele 2 homozygotes may lead to a sharp increase of inflammation, which negatively influences the recovery or remodelling. Further investigation is necessary to confirm these findings.

### Tumour necrosis factor −308 G>A polymorphism and the secondary phase of exercise-induced muscle damage

Tumour necrosis factor (formerly known as tumour necrosis factor-α) is a pro-inflammatory cytokine with short half-life and low circulating levels (Reid and Li 2001; Pedersen 2011) and is associated with the occurrence of metabolic disorders (Borst 2004). Plomgaard et al. (2005) have shown that TNF infusion in healthy individuals alters insulin signalling transduction and subsequently induces insulin resistance in skeletal muscle. Like IL-1β, systemic TNF concentration does not change or is only slightly increased after intense exercise (Peake et al. 2015). However, local expression of TNF within the skeletal muscle is significantly elevated after exercise (Peake et al. 2015). Tumour necrosis factor is associated with up-regulation of catabolic pathways and suppression of protein synthesis in skeletal muscle (Ling et al. 1997), mediated by NF-κB, which stimulates the ubiquitin–proteasome pathway (Reid and Li 2001). This is in line with Tiainen et al. (2012), who have shown that high plasma levels of TNF are associated with reduced physical performance in men. Furthermore, intravenous infusion of TNF in rats led to a significant drop in systemic IGF-I and IGF-binding proteins 3 levels, suggesting a negative influence of TNF on the IGF system (Llovera et al. 1998).

The minor A-allele of the rare *TNF* −308 (rs1800629) SNP is associated with increased plasma TNF concentration (Karimi et al. 2009) and with impaired improvement of physical performance in older women following physical activity (Pereira et al. 2013). Presumably, the A-allele is a stronger activator of TNF transcription than the G-allele (Wilson et al. 1997). To the best of our knowledge, only one study has investigated an association between the *TNF* −308 (G>A) SNP and its association with exercise-induced muscle damage. Interestingly, carriers of the A-allele showed a non-significant (*P* = 0.06) blunting of elevated plasma CK following eccentric exercise (Yamin 2009; Yamin et al. 2008). However, no AA homozygotes were included in this investigation. The *TNF* −308 A-allele was associated with higher plasma TNF concentration and impaired improvements in physical fitness following chronic exercise in older populations, while in young, healthy individuals, A-allele carriers demonstrated blunted CK activity in the blood after eccentric exercise. However, CK activity was measured at the peak activity 96 h post-exercise in Yamin et al. (2008). The blunted CK activity of *TNF* −308 A-allele carriers in the study by Yamin et al. (2008) might not be attributed to the muscle damage itself but may be caused by attenuated remodelling, such as myoblast fusion which is accompanied by CK activity (Zalin 1972). Due to the fact that membrane damage might be repaired in a short time (Bansal et al. 2003), other mechanisms should be considered for the prolonged leakage of CK. Elevated TNF attenuates myoblast fusion and differentiation which might impair the regeneration of the muscle (Stewart et al. 2004). Subsequently, carriers of the *TNF* −308 A-allele might have a higher susceptibility to muscle atrophy and sarcopenia due to the impaired ability of muscle remodelling. However, Lappalainen (2009) has indicated some technical limitations of the assay which might have influenced the data interpretation of Yamin et al. (2008). Further studies are needed, which investigate a potential association between the *TNF* −308 SNP and other muscle damage markers.

### Interleukin-6 −174 G>C polymorphism and the secondary phase of exercise-induced muscle damage

Interleukin-6 (IL-6) modulates the release of different cytokines, such as of TNF and IL-1Ra (Steensberg et al. 2003; Starkie et al. 2003). The human *IL6* gene is mapped to chromosome 7p21–24 with a 303 bp upstream promoter (Fishman et al. 1998). Interleukin-6 plasma concentration is affected by exercise duration and intensity (Fischer et al. 2004), and the amount of muscle mass involved (Ostrowski et al. 2000), particularly during weight-bearing exercise (Catoire and Kersten 2015). Eccentric exercise induces a delayed peak and a slower decrease of plasma IL-6 after exercise in comparison to other modes of exercise, such as running (Fischer 2006; Pedersen and Fischer 2007). According to McKay et al. (2009), IL-6 may play a role as an important signalling molecule associated with satellite cell proliferation after strenuous exercise. Furthermore, damaged extracellular matrix might have an effect on IL-6 expression, as IL-6 is involved in collagen synthesis (Andersen et al. 2011). These findings suggest that the different circulating IL-6 timescale of prolonged but non-damaging exercise and of eccentric exercise occurs due to a different source and function of IL-6 expression. Whilst muscle fibres, peritendinous connective tissue (Langberg et al. 2002) and adipose tissue (Holmes et al. 2004) all express and release IL-6 into the circulation without activating pro-inflammatory pathways (Pedersen 2011), eccentric exercise might induce more local IL-6 expression within the skeletal muscle with pro-inflammatory properties (Nieman et al. 1998, 2000). The delayed peak of plasma IL-6 concentration after strenuous eccentric exercise might occur due to release into the circulation following the mechanical tearing of the sarcolemma and opening of stretch-activated channels due to exertional muscle damage.

A functional −174 G>C SNP (rs1800795) has been detected in the promoter region of the *IL6* gene. The frequency distribution of the G-allele ranges between 45 and 100 % in the worldwide population (Borinskaya et al. 2013) and it is associated with an increased plasma IL-6 response in healthy people (Bennermo et al. 2004; Fishman et al. 1998; Pereira et al. 2011). The −174 G-allele might affect the glucocorticoid receptor and elevate the transcriptional activation due to its close positioning with the receptor (Yamin et al. 2008; Rein et al. 1995). This *IL6* SNP shows a somewhat ambiguous picture: according to Ruiz et al. (2010), both GG and GC genotypes are more frequent in elite power athletes compared to endurance athletes and to non-athletes. There was no difference between endurance athletes and the control group, which is in the line with the findings of Yamin et al. (2008). In young individuals, C-allele carriers of the *IL6* SNP presented higher CK values following eccentric exercise compared with GG homozygotes (Yamin et al. 2008; Yamin 2009). In power-orientated sports, which are associated with muscle damage during training or competition, GG homozygotes might have benefits with faster recovery and elevated satellite cell proliferation in the long term. However, Deuster et al. (2013), who did not observe any association between this *IL6* SNP and exertional rhabdomyolysis, challenge this conclusion.

Ageing-related declines in physical function are associated with chronically elevated systemic IL-6 concentration (Ershler and Keller 2000; da Cunha Nascimento et al. 2015). However, Walston et al. (2005) could not confirm any association between *IL6* genotypes and serum IL-6 in older women. Furthermore, in the study of Funghetto et al. (2013), in older obese women, plasma CK integral (area under the curve of CK between the different time points) values were lower and IL-6 integral values were higher for carriers of the C-allele after eccentric exercise. However, there was only a moderate increase in plasma CK concentration and no change in IL-6 concentration, probably resulting from the relatively low intensity of the eccentric exercise protocol used. Of note, the interaction between the −174 G>C SNP and obesity seems to be a complex one (Joffe et al. 2013). Linkage disequilibrium of this −174 G>C SNP with several other SNPs on the *IL6* gene cannot be excluded (Qi et al. 2007). In diseased, obese and older populations with chronically elevated circulating IL-6, an increased IL-6 response might be harmful after eccentric exercise (Funghetto et al. 2013; Bennermo et al. 2004).

In summary, the pattern of circulatory IL-6 and CK levels in association with the *IL6* −174 G>C SNP appears to be diametrically opposed. It might be that an elevated IL-6 response and lower CK levels associated with the G-allele are beneficial due to increased IL-6 production of macrophages (Patel et al. 2010) and satellite cell proliferation (McKay et al. 2009) in a healthy population following eccentric exercise (Yamin et al. 2008). However, the G-allele might have a negative effect in those presenting with chronic low-grade systemic inflammation. Without knowing the actual source of IL-6 expression and its subsequent pro- or anti-inflammatory effect, cumulative plasma IL-6 concentration is probably an inaccurate biomarker of muscle damage (Pedersen and Febbraio 2008). The influence of the *IL6* −174 G>C SNP is not fully clear and needs further investigation, particularly in conjunction with both local and circulatory measures of IL-6 expression/concentration.

### Chemokine ligand 2 and chemokine receptor type 2 polymorphisms and the secondary phase of exercise-induced muscle damage

Like interleukin-6, the chemokine (C–C motif) ligand-2 (CCL2), also known as monocyte chemoattractant protein 1 (MCP1), can be classified as an exercise factor, as it mediates systemic changes induced by chronic exercise training (Catoire and Kersten 2015). Monocyte chemoattractant protein 1 receptor (*CCR2*) is one of the major receptors, which binds CCL2, beside CCL7 and CCL13 (Harmon et al. 2010). CCL2 is mainly expressed within the interstitial space between myofibres following muscle damaging exercise, and is co-localised with macrophages and satellite cells in the muscle (Hubal et al. 2008). Concentric exercise does not influence local *CCL2* expression (Hubal et al. 2008). However, in line with the findings of Warren et al. (2005), that *Ccr2*-knockout mice have shown impaired regeneration, inflammation, and fibrotic response following freeze injury, a strong interaction between *CCL2/CCR2* and the immune response after muscle damage is suggested (Hubal et al. 2008; Yahiaoui et al. 2008). Interestingly, whilst local *CCL2* mRNA expression further increased after a second bout of eccentric exercise in comparison to the first bout (Hubal et al. 2008), systemic response of CCL2 decreased after repeated downhill running (Smith et al. 2007).

Hubal et al. (2010) investigated several *CCL2/CCR2* SNPs in association with exercise-induced muscle damage in the elbow flexor muscles. Following strenuous exercise, the T-allele of the *CCL2* rs3917878 (C>T) SNP was associated with a delayed recovery of maximum strength in men and a higher CK response in women (Hubal et al. 2010). C-allele carriers of the *CCR2* (rs3918358) SNP showed a delayed recovery of strength in females, and the C-allele of the *CCR2* (rs1799865) SNP increased soreness in both genders (Hubal et al. 2010). The significant differences between the alleles of these three SNPs occurred 4–10 days following exertional muscle damage, confirming the action pattern of CCL2/CCR2 in muscle repair/regeneration. Furthermore, the GG genotype of the *CCL2* gene variant (rs2857656), for which significant differences were found in pre-exercise maximum strength compared to the major C-allele (Harmon et al. 2010), was associated with the magnitude of muscle injury in professional soccer players (Pruna et al. 2013). According to Hubal et al. (2010), there were moderate associations between *CCL2/CCR2* genotypes and baseline CCL2 activity (as a product of CCL2 expression and the availability of CCR2). Higher CCL2 activity might be an advantage in the recovery period following muscle damage in healthy individuals due to its ability to serve as a chemoattractant to macrophages and its possible activation of satellite cell proliferation (Yahiaoui et al. 2008). However, further investigation is needed to identify the potential molecular mechanisms underpinning the influence of each of these SNPs in changing CCL2 activity in response to muscle damaging exercise in elderly and obese people, in whom chronic systemic inflammation is already an issue.

### Osteopontin −66 T>G polymorphism and the secondary phase of exercise-induced muscle damage

The extracellular matrix protein and pro-inflammatory cytokine osteopontin (also known as secreted phosphoprotein 1) is expressed in numerous cell types including skeletal muscle (Kahles et al. 2014; Zanotti et al. 2011; Giachelli et al. 1998). Whereas the earliest studies suggested that it had a central role in bone remodelling (Rodan 1995), subsequent studies suggest that osteopontin has also a role as a chemoattractant for macrophages (Hirata et al. 2003), and possibly neutrophils (Yang et al. 2014). Osteopontin is virtually undetectable in resting skeletal muscle but, after induced muscle damage in mice, osteopontin expression is elevated 100-times compared to baseline transcription levels (Hoffman et al. 2013; Hirata et al. 2003).

A common SNP in the transcriptional promoter of the osteopontin gene (−66 T>G, rs28357094), which overlaps a specificity protein-1 transcription factor-binding site, results in different phenotypic characteristics (Barfield et al. 2014). The minor G-allele is associated with an 80 % reduction in osteopontin gene expression in vitro (Giacopelli et al. 2004; Barfield et al. 2014) and with a 17 % increase in baseline upper arm muscle volume in women (Hoffman et al. 2013). Surprisingly, this increased muscle volume did not influence muscle strength (Hoffman et al. 2013). After exercise-induced muscle damage, women carrying the G-allele revealed significantly elevated muscle swelling, increased loss of muscle strength (Barfield et al. 2014) and CK values were elevated in two women with the rare GG genotype (Hoffman et al. 2013). In contrast, the G-allele was linked with less grip strength and with more rapid progression in patients with Duchenne muscular dystrophy (Pegoraro et al. 2011). Further investigations of Barfield et al. (2014) revealed several enhancer sequences on the osteopontin gene promoter for multiple steroid hormone-binding sites (i.e. oestrogen receptor, glucocorticoid receptor, vitamin D receptor and a potential NF-κB binding site). Oestrogen hormone treatment of modified human myoblasts with the allele-specific osteopontin promoters has shown that the human myoblasts with the transfected G-allele promoter revealed a threefold increase in osteopontin gene expression, whereas the T-allele construct was unaffected by oestrogen treatment. From this, we can infer that there may be an allele-specific interaction between the oestrogen enhancer and the more proximal specificity protein-1 transcription factor site leading to a hypothetical model for sexual dimorphism (Barfield et al. 2014). Thus, women with the G-allele seem to be more susceptible to muscle damage. Likewise, a similar allele-specific interaction between the NF-κB or glucocorticoid binding site and the specificity protein-1 transcription factor site might explain the association between the G-allele and Duchenne muscular dystrophy. Barfield et al. (2014) suggest that chronic inflammation might lead to an augmentation of the pro-inflammatory response, which accelerates the progress of the disease. However, the study of Barfield et al. (2014) has several limitations. TT genotype has shown a similar loss of force over time in both the exercised and non-exercised arm following exertional muscle damage. In addition, due to the low number of volunteers (*n* = 6) who completed the eccentric exercise intervention, further investigations are needed to replicate and verify these findings.

## Skeletal muscle remodelling following exercise-induced muscle damage

**Fig. 4:**
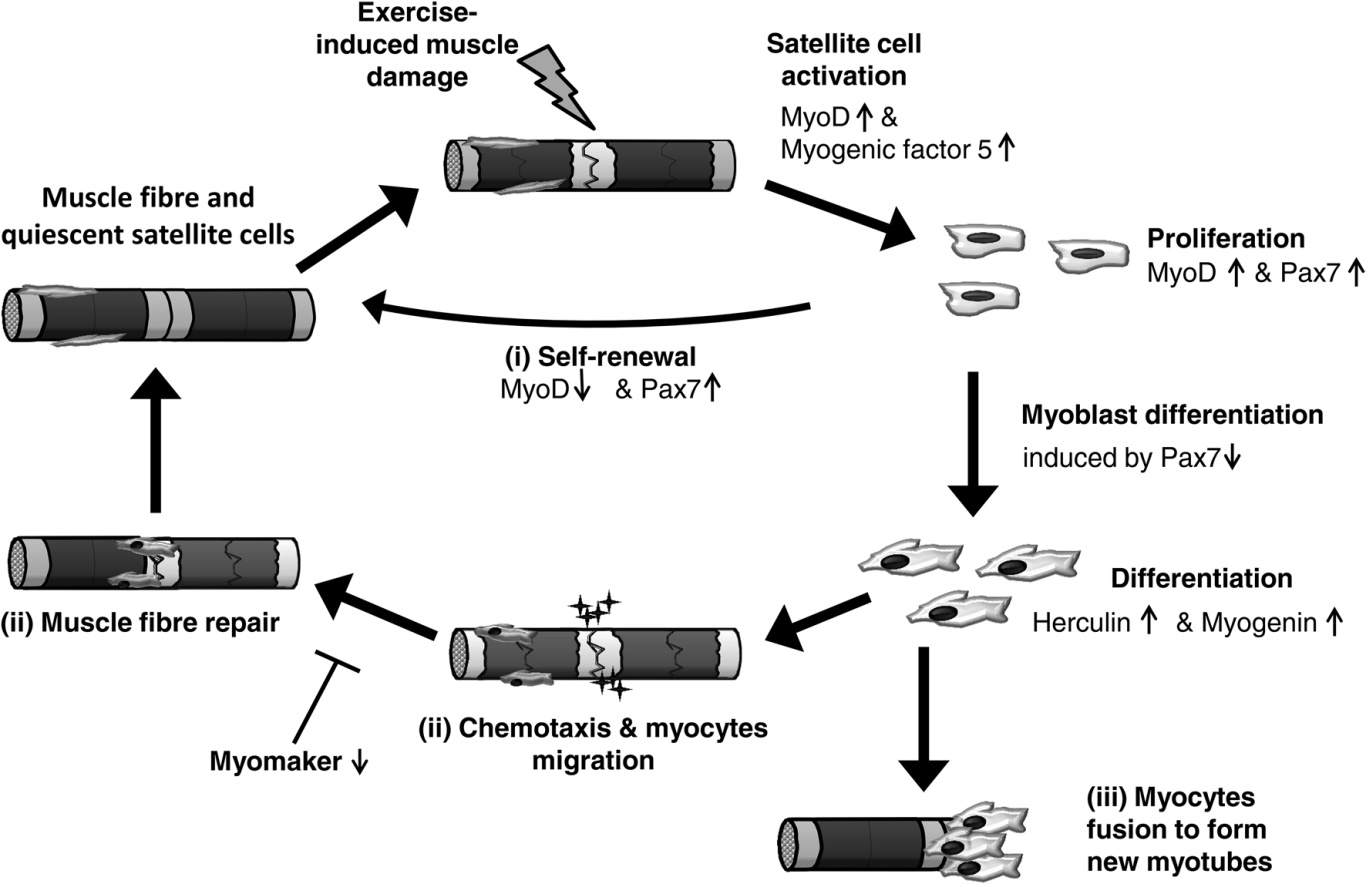
The cycle of skeletal muscle fibre regeneration following exercise-induced muscle damage. This cycle is mediated by satellite cells, which are activated following stressful physiological conditions such as exercise-induced muscle damage (Grobler et al. 2004). Activated satellite cells initially up-regulate two different myogenic regulatory factors, MyoD and myogenic factor-5 and, during the proliferation, paired box protein 7 (Pax7). If satellite cells return to quiescence and restore the population of satellite cells, MyoD will be down-regulated (*i*). However, subsequent cell differentiation is accompanied with down-regulation of Pax7/3. During this early differentiation stage, herculin and myogenin are up-regulated. Myoblasts differentiate into myocytes and then eventually migrate to the site of injury and support the repair process by increasing the nuclei-to-cytoplasm ratio (*ii*). Different chemotactic gradients, including a large number of chemokines, support the migration to the region of injury. A recent investigation in mice revealed that the absence of myomaker, which is expressed on the cell surface of myoblasts, leads to inhibition of myoblast fusion (Millay et al. 2013). Alternatively, the myocytes fuse with other myogenic cells to form myotubes, thus generating new fibres to replace damaged myofibres (*iii*). Figure adapted from Tidball (2011) and Al-Shanti and Stewart (2009)

Skeletal muscle regeneration is a complex process that is mediated by satellite cells, and in which several factors are activated to regulate muscle remodelling (Kurosaka and Machida 2012). Satellite cells are mononucleated muscle stem cells and are located on the outer surface of the muscle fibre, between the basal lamina and sarcolemma (Hawke and Garry 2001). Usually, satellite cells remain quiescent but are activated following damage (Fig. 4) (Chambers and McDermott 1996; Grobler et al. 2004). They proliferate 24–48 h later and then do one of three things: (1) return to quiescence and restore the population of satellite cells; (2) migrate to the site of injury and support the repair process by increasing the nuclei-to-cytoplasm ratio; (3) fuse with other myogenic cells to form myotubes, thus generating new fibres to replace damaged myofibres (Hawke and Garry 2001; Grobler et al. 2004; Tidball and Villalta 2010; Sharples and Stewart 2011).

Macrophages are essential, not only for removing tissue debris, but also in the activation of satellite cells. M1 macrophages provoke myoblast proliferation (Arnold et al. 2007; Cantini et al. 2002) and, together with neutrophils, they attract satellite cells to the site of injury by releasing TNF (Torrente et al. 2003). M2 macrophages stimulate the differentiation of satellite cells into mature myofibres (Arnold et al. 2007), and in vitro studies indicate that macrophages support differentiation through ultimate increases in myogenin expression (Cantini et al. 2002). Activated satellite cells initially up-regulate two different myogenic regulatory factors, MyoD and myogenic factor-5 (Smith et al. 1994). In the period of proliferation, the satellite cells express paired box protein 7 (Pax7) and MyoD but those that return to quiescence to maintain the satellite cell pool only express Pax7 (Tedesco et al. 2010; Al-Shanti and Stewart 2009). However, subsequent down-regulation of Pax7/3 induces cell differentiation. The satellite cells exit the cell cycle and enter the early differentiation stage where myogenic factor 6 (herculin) and myogenin are up-regulated (Zammit 2008; Wahl et al. 2008; Le Grand and Rudnicki 2007). Myoblasts differentiate into myocytes and then eventually fuse and form multinucleated myofibres (Le Grand and Rudnicki 2007). Recent investigations revealed that MyoD and myogenin induce myomaker gene transcription (Millay et al. 2013, 2014). The absence of myomaker, which is expressed on the cell surface of myoblasts, leads to inhibition of myoblast fusion in mice (Millay et al. 2013). However, more information is required to explain the roles of myomaker in muscle regeneration and recovery following muscle damaging exercise.

The extracellular matrix provides structural and biochemical support to contractile tissue and it is associated with the inflammatory response and satellite cell activation (Hyldahl and Hubal 2014; Kjær 2004). Activated satellite cells migrate to the site of injury along the basal lamina (Hughes and Blau 1990), a process that is facilitated by the basal lamina components (i.e. collagen IV, laminin-2 and nidogens) (Goetsch and Niesler 2011). Components of the extracellular matrix (collagen I and III, fibronectin and other extracellular matrix molecules) provide a temporary scaffold to support the migration of the activated progenitor cells (Goetsch et al. 2013). Different chemotactic gradients, including a large number of chemokines, also support the migration from the niche towards the site of myotrauma, and some of these chemokines are released from the extracellular matrix itself (Griffin et al. 2010; Goetsch et al. 2013). Furthermore, there is evidence that synthesis of type I, III and probably IV collagen within the endomysium and the perimysium increase after contraction-induced damage (Mackey et al. 2004; Koskinen et al. 2001).

Chronic low-grade systemic inflammation (i.e. elevated levels of circulating pro-inflammatory cytokines), is a common observation in older people (Conceição et al. 2012; Franceschi et al. 2007). In this context, basal circulating cytokines (e.g. TNF and IL-6) and myostatin were found to correlate inversely with grip strength of older men (Patel et al. 2014). Although the mechanism for this inverse relationship is still unclear, it is possible that pro-inflammatory cytokines interfere with satellite cell differentiation, accelerate muscle protein degradation and inhibit muscle protein synthesis, leading to reduced muscle mass and strength. This would also result in slower repair and reduced adaptation of older skeletal muscle to resistance exercise (Peake et al. 2010). Indeed, Dreyer et al. (2006) have counted the numbers of satellite cells per muscle fibre 24 h after a single bout of maximal eccentric exercise. Although both young and older men demonstrated an increase in satellite cell numbers, the response was significantly blunted in the older population. However, it is still a matter of debate, if the number of satellite cells changes during the ageing process, and whether this is the main cause for what has been coined anabolic resistance in the elderly.

### Gene polymorphisms of the insulin-like growth factor family and the remodelling fsignificant gain in muscle cross-sectional areaollowing exercise-induced muscle damage

The complex process of remodelling is influenced by growth factors including insulin-like growth factor-I (IGF-I) and IGF-II (Duan et al. 2010). In addition to IGF-I (*IGF1*) and IGF-II (*IGF2*), the IGF system consists of several IGF-binding proteins, the insulin receptor, and cell surface receptors such as the IGF-I receptor and the IGF-II receptor (Wang et al. 2015). This system promotes satellite cell differentiation and proliferation (O’Dell and Day 1998; Florini et al. 1996; Stewart and Rotwein 1996a, b; Stewart et al. 1996) and is thought to play an important role during exercise-induced muscle hypertrophy (Sharples and Stewart 2011; Matheny et al. 2009). For example, transgenic mice overexpressing Igf-I in skeletal muscle revealed a significant gain in muscle cross-sectional area in comparison with wild type mice following chronic muscle overload (Paul and Rosenthal 2002). Inactivation of the type 1 Igf receptor inhibits the presence of newly formed nuclei in exercised transgenic mice (Fernández et al. 2002; Jiao et al. 2013; Wilson et al. 2003), while maintaining local IGF-I concentration is considered crucial for maintaining muscle mass and strength with advancing age (Barton-Davis et al. 1998; Musarò et al. 2001).

Besides their role in hypertrophy, IGFs are crucial in muscle regeneration following exercise or muscle injury (Jiao et al. 2013; Mackey et al. 2011). Insulin-like growth factor-I acts mainly in an autocrine and paracrine manner to stimulate satellite cells to proliferate and differentiate. Different isoforms [IGF-IEa, IGF-IEb (in rat) and IGF-IEc (in human)] of IGF-I are associated with muscle damage and regeneration. Insulin-like growth factor-IEb and IGF-IEc are also known as mechano-growth factor, because the mRNA is expressed in response to overload or damage in skeletal muscle. The expression of mechano-growth factor is enhanced shortly after muscle damage, which subsequently promotes satellite cell activation (Hill and Goldspink 2003). Afterwards, increased expression of IGF-IEa elevates myoblast fusion (Yang and Goldspink 2002; Jiao et al. 2013). Mechano-growth factor also promotes the activity of cytoplasmic superoxide dismutase, thus protecting against ROS during the inflammatory response to muscle damaging exercise (Dobrowolny et al. 2005). Both IGF-I and IGF-II mRNA increase during myoblast differentiation, but presumably autocrine IGF-II is the predominant myogenic factor during differentiation due to its enhanced expression, whilst IGF-II is probably elevated to suppress IGF-I gene expression via the mTOR pathway (Jiao et al. 2013; Wilson et al. 2003). Marsh et al. (1997) have also shown an age-dependent decline of *IGF2* gene expression following muscle damage in rats.

As far as we are aware, only Devaney et al. (2007) have tested the association between IGF SNPs and exercise-induced muscle damage. Several different SNPs were investigated, as the *IGF2* gene region consists of three genes: *IGF2, IGF2* anti sense (*IGF2AS)*, and the insulin gene (Lee et al. 2005). The following SNPs: *IGF2* (17200 G>A, rs680); *IGF2* (13790 C>G, rs3213221); *IGF2AS* (1364 A>C, rs4244808); *IGF2AS* (11711 G>T, rs7924316), were significantly associated with exercise-induced muscle damage. Besides an association between the *IGF2* 17200 (G>A, rs680) and *IGF2* 13790 (C>G, rs3213221) SNPs and soreness (after 3 and 4 days), and CK activity in the blood (both after 7 days) following muscle damaging exercise, every *IGF2* SNP investigated was associated with strenuous exercise-induced muscle strength loss in men. Only the *IGF2AS* 1364 (A>C, rs4244808) SNP was associated with strength loss immediately after exertional muscle damage in both men and women. In contrast, carriers of the insulin gene 1045 (C>G, rs3842748) SNP have shown an increased CK activity 10 days after exercise-induced muscle damage only in women.

Varying IGF-I or IGF-II levels potentially caused by these SNPs could modulate satellite cell activation and differentiation. For instance, the *IGF1* cytosine adenine-repeat SNP located in the promoter region of the IGF-I gene is believed to change circulating IGF-I levels but the evidence is equivocal (Vaessen et al. 2001; Rosen et al. 1998; DeLellis et al. 2003; Allen et al. 2002). While Vaessen et al. (2001) suggest IGF-I levels are increased by these SNPs, other investigations found a decrease (Rosen et al. 1998) or no difference in IGF-I levels (Allen et al. 2002; DeLellis et al. 2003).

It is remarkable that several SNPs of *IGF2* were associated with a loss of muscle strength directly after exertional muscle damage, in particular in men. It seems there must be another process, whereby the *IGF2* gene is involved in the response to muscle damaging exercise separately from regeneration and differentiation. Here, we would like to highlight a new hypothesis. Insulin-like growth factor I also plays an important role in the regulation of protein synthesis, including collagen and myofibrillar protein. Local IGF-IEa and IGF-IEc mRNA expression is positively correlated with musculotendinous mRNA expression of *COL1A1/3A1* (Boesen et al. 2013; Doessing et al. 2010) and may subsequently increase collagen synthesis in the extracellular matrix (Hansen et al. 2013). Lower circulating IGF-I levels induced by IGF SNPs may negatively influence the stability of the extracellular matrix. Therefore, a subsequent loss in the lateral transmission of force between adjacent muscle fibres might occur, which could be the source of the decrease in maximum strength observed immediately after strenuous exercise. Although, to the best of our knowledge, no direct effect of IGF-II concentration on human extracellular matrix protein synthesis is known, Keller et al. (1999) has shown that local Igf-II expression increases after injury in murine muscle. It is therefore possible that IGF-II is linked with exercise-induced muscle damage in human muscle, and possibly with extracellular matrix integrity. A direct or indirect influence of IGF-II level on extracellular matrix integrity would, at least in part, explain the significant strength loss after muscle damaging exercise and the association of the *IGF2* 13790 (C>G, rs3213221) SNP with the degree of injury in soccer players (Pruna et al. 2013).

## Additional gene polymorphisms associated with exercise-induced muscle damage

The following gene polymorphisms have been associated with exercise-induced muscle damage. However, further investigation is necessary to attribute these polymorphisms to a specific phase of exercise-induced muscle damage.

### Angiotensin-I converting enzyme insertion/deletion polymorphism

Angiotensin-I converting enzyme (ACE) has a key role in the interaction between the kallikrein-kinin and the renin-angiotensin systems (Schmaier 2003). Angiotensinogen, which is a precursor protein in the renin-angiotensin system, is produced constitutively and released into the circulation mainly by the liver (Deschepper 1994), and can be cleaved by the protease renin, resulting in the decapeptide angiotensin-I. The dipeptidase ACE converts angiotensin-I to the octapeptide hormone angiotensin-II, which acts as a vasoconstrictor (Munzenmaier and Greene 1996), and induces skeletal muscle hypertrophy in response to mechanical loading (Gordon et al. 2001). Angiotensin-I converting enzyme also cleaves the vasodilator bradykinin (Dendorfer et al. 2001), which supports the increase of arterial blood pressure (Murphey et al. 2000), as well as Substance P, a protein from the tachykinin family that functions as a neurotransmitter (released by group III and IV afferent fibres) (Harrison and Geppetti 2001; Inoue et al. 1998).

The *ACE* insertion/deletion (I/D) polymorphism (rs4646994) was the first gene variation to be investigated in the context of human physical performance-related traits, and is the most investigated in the renin–angiotensin system (Gayagay et al. 1998; Montgomery et al. 1998). The insertion (I) allele of a 287 bp Alu sequence within intron 16 on chromosome 17 is linked to lower ACE activity in serum (Rigat et al. 1990) and in cardiac muscle (Phillips et al. 1993; Danser et al. 1995), and reduced bradykinin degradation (Murphey et al. 2000) compared to carriers of the D-allele. Carriers of the I-allele are associated with greater endurance capacity (Montgomery et al. 1998; Ma et al. 2013), whereas the D-allele is associated with greater muscular strength (Williams et al. 2005), and elite power athlete status (Costa et al. 2009; Nazarov et al. 2001; Woods et al. 2001). However, recent investigations have observed that this distinction is not considered sufficiently specific to detect all the phenotypic effects (Lucia et al. 2005; Rankinen et al. 2000; Thompson and Binder-Macleod 2006).

The association between the *ACE* I/D polymorphism and elite athlete status might be explained by a genotype link with the susceptibility to exertional muscle damage and injury. To the best of our knowledge, only two studies have investigated the influence of the *ACE* I/D polymorphism on contraction-induced damage in humans (Heled et al. 2007; Yamin et al. 2007). Yamin et al. (2007) observed different concentrations of circulatory CK between *ACE* genotypes after eccentric exercise: II homozygotes elicited the highest CK response, whilst DD homozygotes elicited the lowest plasma CK activity after strenuous exercise. This suggests that the I-allele is associated with a greater susceptibility to muscle damage, and the potential mechanism is explained below. However, Heled et al. (2007) could not find any association between *ACE* I/D polymorphism and CK response. The different outcome is probably attributed to the moderate-intensity exercise test and higher activity level and different ethnicities of the participants in the study of Heled et al. (2007). It should be noted that only CK level was investigated in both studies, which is only one of several indirect biomarkers of exercise-induced muscle damage.

In rabbit studies, inhibition of ACE revealed [in combination with neutral endopeptidase inhibitor] elevated muscle damage in a muscle overuse model induced by electrical stimulation every second day with four sessions in total (Song et al. 2014), which is in line with the human findings of Yamin et al. (2007). The muscle damage was accompanied by increased tachykinin, substance P and its preferred receptor neurokinin-1 receptor expression, which suggests that the tachykinin family may play a role in the inflammatory processes and pain (Song et al. 2014; Dousset et al. 2007). Substance P is widely expressed in human cells and tissues of the peripheral and central nervous systems but it is also found in extra neuronal cells and innervated tissues. Substance P and neurokinin-1 receptor have been associated with the inflammatory response in smooth muscle cells and dermal tissues but not in skeletal muscle (Renzi et al. 2000; Luger 2002). However, elevated substance P might result in improved remodelling, as demonstrated in the healing of a rat Achilles tendon (Bring et al. 2012; Steyaert et al. 2010).

In contrast, angiotensin-II is known to be involved in inflammatory process following muscle damage. Blocking of the angiotensin-II receptor type 1 improves regeneration of injured skeletal muscle (Bedair et al. 2008) and suppresses ROS production following strenuous exercise in mice (Sim et al. 2014). Furthermore, nerve growth factor up-regulation through activation of B_2_ bradykinin receptors is strongly associated with increased pain sensitivity (hyperalgesia) (Murase et al. 2010; Babenko et al. 1999). Angiotensin-I converting enzyme D-allele carriers, which have a decreased bradykinin half-life, might have attenuated nerve growth factor expression following exertional muscle damage and therefore a decreased pain sensitivity. Attenuated substance P and bradykinin in the inflammatory process may explain the high frequency of D-allele carriers among elite strength/power athletes (Costa et al. 2009). Athletes with the D-allele might feel less pain and therefore might be able to (1) sustain high-intensity training for longer, (2) reach the limits of their capacity in power/strength related competition (3) or enable them to practise more often due to a decreased sensitivity to pain. In other sport-specific movements, such as short distance swimming (<200 m), it is crucial to sustain a high level of intensity accompanied with exercise-induced muscle burning (Costa et al. 2009; Woods et al. 2001).

Another possibility might be that angiotensin-II indirectly mediates skeletal muscle damage by influencing angiogenesis in response to exercise (Vaughan et al. 2013). It is well known that, in a damaged muscle in the days following eccentric exercise, resting capillary blood flow is elevated and vasodilatation occurs (Rubinstein et al. 1998). According to Vaughan et al. (2013), the capillary density of skeletal muscle is lower in untrained carriers of the *ACE* I-allele compared to DD homozygotes. Lower capillary density might impair the migration of neutrophils and macrophages as well as of the removal of cellular debris, which could negatively affect the extent of muscle damage and possibly muscle remodelling.

### Mitochondrial superoxide dismutase 2 Ala16Val polymorphism

Strenuous exercise results in oxidative stress, which causes structural damage to muscle fibres and stimulates an inflammatory response (Gomez-Cabrera et al. 2008), as discussed in “Genetic variation and the secondary phase of exercise-induced muscle damage”. A higher intracellular concentration of antioxidants within a muscle fibre is thought to protect against the negative impact of ROS (Schoenfeld 2012; Peake and Suzuki 2004). Superoxide dismutase is an antioxidant that protects cells and mitochondria from free radical damage by converting the anion superoxide into hydrogen peroxide (Huang et al. 2000). Inhibition of superoxide dismutase causes the accumulation of superoxide radicals, and can lead to increased damage of mitochondrial membrane and cell apoptosis (Huang et al. 2000). The Ala16Val (rs4880, C>T) SNP of the superoxide dismutase 2, mitochondrial gene (*SOD2*), has been associated with muscle damage susceptibility. The T-allele is associated with reduced mitochondrial superoxide dismutase efficiency against oxidative stress (Shimoda-Matsubayashi et al. 1996). Akimoto et al. (2010) demonstrated that trained runners of TT genotype had an increased plasma CK concentration after racing 4–21 km. This is in line with Ahmetov et al. (2014), who revealed that TT carriers of the mitochondrial superoxide dismutase gene were under-represented in power and strength athletes compared to controls and athletes of low-intensity sports, such as curling players and shooters. Interestingly, in the study of Ben-Zaken et al. (2013), the frequency of the C-allele was significantly higher in both endurance and power athletes in comparison to the control group. At first glance, these studies seem to be inconsistent with one another. On closer inspection, both studies recruited different types of participants. In the study of Ahmetov et al. (2014) participants covered a wide range of different sports, whereas in Ben-Zaken et al. (2013), only track and field related athletes participated: 100 and 200 m sprinters and long jumpers (power athletes); 5000 m and marathon runners (endurance athletes). These track and field athletes perform sport-specific movements that is accompanied by stress to the musculoskeletal system through repeated eccentric muscle contractions performed over long periods of time, which leads to muscle damage. The inflammation accompanying this damage potentially produces more oxidative stress than the endurance sports (e.g. swimming) in the study by Ahmetov et al. (2014). Therefore, the T-allele might impair the protection against oxidative stress due to the lower efficiency of the mitochondrial superoxide dismutase gene. This may indicate that there is a relationship between this SNP and level of athletic performance in sports with a potential risk of muscle damage. Unfortunately, no study has tested the effect of the mitochondrial superoxide dismutase SNP on exercise-induced muscle damage over the course of time. This could provide insight into the influence of the mitochondrial superoxide dismutase C>T SNP on the secondary phase of muscle damage and the subsequent remodelling.

### Solute carrier family 30 member eight C>T polymorphism

Ageing is often accompanied by insulin resistance due to reduced habitual physical activity, a reduction in muscle mass and an increase in adipose tissue (Dela and Kjaer 2006; Dela et al. 1996). Type 2 diabetes mellitus is associated with disturbed zinc homeostasis and down-regulation of the solute carrier family 30 (zinc transporter) member eight, the product of the *SLC30A8* gene (Somboonwong et al. 2015). Solute carrier family 30 member eight is mainly expressed in pancreatic islet beta cells and it transports zinc from the cytoplasm into intracellular vesicles, which is crucial for insulin crystallisation, storage, and secretion (Cheng et al. 2015; Lemaire et al. 2009; Chimienti et al. 2006). The C-allele of the nonsynonymous *SLC30A8* (C>T) SNP (rs13266634) is strongly associated with type 2 diabetes mellitus risk, in particular in European and Asian populations but not in African populations (Cheng et al. 2015). This *SLC30A8* R325W SNP is associated with, amongst others, decreased fasting systemic insulin and attenuated insulin secretion in response to glucose intake (Staiger et al. 2007; Sprouse et al. 2014; Kirchhoff et al. 2008).

In recent years, there has been an increase in the number of investigations regarding insulin resistance and muscle function in people without type 2 diabetes mellitus. Insulin resistance is not only associated with lower force and muscle mass in young and old individuals with diabetes (Andreassen et al. 2009; Andersen et al. 2004), but also in both young (Gysel et al. 2014) and older (Barzilay et al. 2009) healthy people. Insulin signalling increases blood flow and protein synthesis at rest, and suppresses the breakdown of proteins after resistance exercise, thus improving net muscle protein balance in particular with amino acid delivery and availability (Biolo et al. 1999; Fujita et al. 2006). Furthermore, exercise-induced muscle damage has been associated with impaired glycogen synthesis (Costill et al. 1990) and reduced glucose uptake (Nielsen et al. 2015; Asp et al. 1996), probably due to muscle damage reducing muscle insulin sensitivity (Kirwan et al. 1991). This could be due to increased TNF expression attenuating insulin signalling transduction, subsequently inducing insulin resistance in skeletal muscle (Plomgaard et al. 2005) and suppressing the activation of glucose transporter type 4 in muscle fibres (Asp et al. 1995).

Sprouse et al. (2014) reported that the TT genotype of the *SLC30A8* SNP was associated with lower biomarkers of muscle damage (reduced soreness, strength loss and plasma CK and myoglobin levels) following eccentric contractions of the elbow flexor muscles in men. By increasing the catabolic pathway, lower insulin levels can lead to a negative net protein balance (Woolfson et al. 1979; Sacheck et al. 2007). Therefore, carriers of the *SLC30A8* C-allele might need longer times to recover from strenuous exercise. Further studies should investigate if *SLC30A8* genotype-dependent insulin production is associated with the acute and chronic adaptations to resistance exercise, with regard to muscle protein synthesis and muscle hypertrophy, respectively.

## Discussion

Exercise-induced muscle damage provokes a prolonged loss of muscle strength, and both elevated soreness and circulating muscle-specific protein levels. The grade and actual time-course of strength loss, soreness and of the inflammation response after exercise is variable. Several factors that are well documented can influence the response to muscle damaging exercise, such as exercise mode, intensity or duration (Smith et al. 1989), micro nutrition (Owens et al. 2014; Bhat and Ismail 2015; Barker et al. 2013) and muscle (group) intervention (Clarkson and Hubal 2002). Nevertheless, within-study variability is often seen in response to strenuous exercise (Nosaka and Clarkson 1996).

Several studies have reported differences in SNP-specific gene activity resulting in different expression of the coding proteins, which may influence the susceptibly to exercise-induced muscle damage (Seto et al. 2011). Individuals, who are high responders to exercise-induced muscle damage (i.e. demonstrate a greater loss of muscle strength and higher circulating levels of CK or myoglobin) might have a higher predisposition to injury (Kibler et al. 1992; Clansey et al. 2012). This is in line with the observation that history of one type of muscle injury increases the risk of developing other types of muscle injuries (Orchard 2001; Freckleton and Pizzari 2013). The same principle may apply to high responders to exercise-induced muscle damage in a squad of athletes performing the same exercise training together. High responders, who might need a longer recovery time after a strength training intervention in comparison to others in the same squad, might have a higher potential for musculotendinous injuries due to overtraining. Both presumptions may result in an increased dropout rate of athletes with specific genotype profiles due to higher rates of (overtraining) injury extending over several years (Kibler et al. 1992). It would be interesting to investigate if a high responder to exercise-induced muscle damage is a low/high responder to chronic resistance training.

Eccentric resistance exercise training is a potent method of inducing muscle hypertrophy (Seynnes et al. 2007; Norrbrand et al. 2008), and may therefore be prescribed to older people to counter sarcopenia (Reeves et al. 2004; Morse et al. 2007). However, the increased susceptibility to muscle damage following eccentric exercise in older people (Ploutz-Snyder et al. 2001) might lead to an increased risk of over-use injuries and impaired recovery from injury (Brooks and Faulkner 1990; McArdle et al. 2004). Furthermore, if certain older individuals are genetically predisposed to experience relatively more exercise-induced muscle damage than age-matched individuals with a protective genetic profile, these people are at an even greater risk of injury. Thus, a different form of exercise may be more appropriate for these individuals. The identification of both young and older high-risk individuals would, therefore, allow more personalised exercise prescriptions to help reduce the risk of injuries and maintain/improve quality of life.

Association studies can potentially reveal new mechanisms of genes. For instance, several *IGF2* SNPs have been associated with strength loss immediately after muscle damaging exercise, which cannot exclusively be explained by satellite cell differentiation (Devaney et al. 2007). It is interesting that (1) certain genotypes of several *IL6* gene SNPs appear to be beneficial in healthy individuals regarding muscle damage response, but are disadvantageous in chronic disease and ageing; (2) sex-specific genotype associations with exercise-induced muscle damage have been reported (Devaney et al. 2007; Sprouse et al. 2014). Further investigations are necessary to uncover genotype–phenotype interactions and, in particular, the interaction of specific polymorphisms. A specific polygenic profile might help to explain the inter-individual variance in the response to both acute eccentric damaging exercise and chronic strength training.

Moreover, the *ACTN3* R577X SNP has been associated with different responses to muscle damaging exercise, according to the mode of damaging exercise. It is likely that stretch–shortening cycle-related movements place different demands on the musculotendinous system compared to exercises, which are performed without stretch–shortening cycles, thus explaining the equivocal findings concerning the association between this SNP and exercise-induced muscle damage (see “Alpha-actinin-3 R577X polymorphism and the initial phase of exercise-induced muscle damage”). Consequently, we recommend that future studies distinguish between exercise-induced muscle damage caused by eccentric contractions with or without stretch–shortening cycles. Furthermore, real-world modes of exercise should be incorporated into studies investigating the genetic association with exertional muscle damage in both young and older people. Not only will this improve our understanding of the mechanisms underpinning the deteriorated response of ageing muscle to exercise, but also it will help in prescribing more practical exercise therapies to poor exercise responders, young or old.

## Conclusions

Understanding the causes and consequences of these genetic associations with exercise-induced muscle damage may eventually allow the identification of individuals, who are at high-risk of developing specific injuries. For instance, those who are genetically more predisposed to muscle damage, and who require longer recover from strenuous exercise, are at greater risk of developing over-use injuries. Knowing how someone is likely to respond to a particular type of exercise would help coaches tailor the training and nutrition of their athletes (moving from a one size fits all to an individualised approach), thus maximising recovery and positive adaptation, and reducing the risk of injury. It would also help general practitioners prescribe personalised exercise medicine to older individuals, who may normally be prescribed resistance type training to counter the effects of sarcopenia, but are already at a higher risk of suffering from exercise-induced muscle damage due to chronically elevated systemic inflammation.

## Abbreviations

ACE: Angiotensin-I converting enzyme
*ACTN3*: Gene that encodes the α-actinin-3 protein
CCL2: Chemokine (C–C motif) ligand-2
CCR2: Chemokine (C–C motif) receptor type-2
CK: Creatine kinase
*COL*: Gene that encodes the collagen protein
IGF: Insulin-like growth factor
IL: Interleukin
mRNA: Messenger ribonucleic acid
MyoD: Myogenic differentiation factor
NF-κB: Nuclear factor kappa-light-chain-enhancer of activated B cells
Pax7: Paired box protein-7
ROS: Reactive oxygen species
*SLC30A8*: Gene that encodes the solute carrier family 30 (zinc transporter) member eight protein
SNP: Single nucleotide polymorphism
TNF: Tumour necrosis factor
